# Inactivation of *ancV1R* as a Predictive Signature for the Loss of Vomeronasal System in Mammals

**DOI:** 10.1093/gbe/evaa082

**Published:** 2020-04-21

**Authors:** Zicong Zhang, Masato Nikaido

**Affiliations:** e1 Department of Computational Intelligence and Systems Science, Tokyo Institute of Technology, Yokohama, Kanagawa, Japan; e2 School of Life Science and Technology, Tokyo Institute of Technology, Meguro-ku, Tokyo, Japan

**Keywords:** vomeronasal organ, ancV1R, pheromone, mammal, pseudogene

## Abstract

The vomeronasal organ (VNO) plays a key role in sensing pheromonal cues, which elicits social and reproductive behaviors. Although the VNO is highly conserved across mammals, it has been lost in some species that have evolved alternate sensing systems during diversification. In this study, we investigate a newly identified VNO-specific gene, *ancV1R*, in the extant 261 species of mammals to examine the correlation between genotype (*ancV1R*) and phenotype (VNO). As a result, we found signatures for the relaxation of purifying selection (inactivating mutations and the elevation of d*N*/d*S*) on *ancV1R*s in VNO-lacking mammals, such as catarrhine primates, cetaceans, the manatees, and several bat lineages, showing the distinct correlation between genotype and phenotype. Interestingly, we further revealed signatures for the relaxation of purifying selection on *ancV1R* in true seals, otters, the fossa, the owl monkey, and alcelaphine antelopes in which the existence of a functional VNO is still under debate. Our additional analyses on *TRPC2*, another predictive marker gene for the functional VNO, showed a relaxation of purifying selection, supporting the possibility of VNO loss in these species. The results of our present study invite more in-depth neuroanatomical investigation in mammals for which VNO function remains equivocal.

## Introduction

The vomeronasal organ (VNO) is a chemosensory structure found in terrestrial vertebrates that is anatomically separated from the main olfactory epithelium (MOE). VNO detects pheromones, whereas MOE mainly detects odorants (Firestein 2001; Brennan and Zufall 2006). Although some overlaps were found between VNO and the MOE in pheromone detection (Mandiyan et al. 2005; Ohara et al. 2009), the VNO plays a major role in eliciting the pheromone-induced sexual and social behaviors. Actually, the surgical ablation of the VNO in mice led to a number of behavioral and physiological deficits in their response to pheromonal cues (Meredith 1986; Wysocki and Lepri 1991). The VNO structure can be found across a broad diversity of mammals because of its importance to pheromone-mediated communication, essential for fitness and survival (Døving and Trotier 1998). However, morphological studies have shown that some mammals do not possess anatomically intact VNOs. For example, cetaceans and sirenians lost the VNO in parallel accompanied by their independent adaptation to fully aquatic lifestyles (Lowell and Flanigan 1980; Switzer et al. 1980; Mackay-Sim et al. 1985; Oelschläiger 1989). Catarrhine primates also lost their functional VNO (Bhatnagar and Meisami 1998) which may be due to their adaptation to a diurnal lifestyle and the acquisition of trichromatic color vision (Dixson 1983). In bats, most species have lost their VNO except for two families (Phyllostomidae and Miniopteridae; Wible and Bhatnagar 1996). These independent losses on each bat lineage are still puzzling. The VNO structure in New World monkeys is still intact, but reductions of some microanatomical components have been suggested (Hunter et al. 1984; Smith et al. 2011). Among semiaquatic pinnipeds, otariids (fur seals, sea lions) and odobenids (walrus) retain the VNO, whereas this organ is lost in phocids (true seals; Mackay-Sim et al. 1985).

**Fig. 1.**
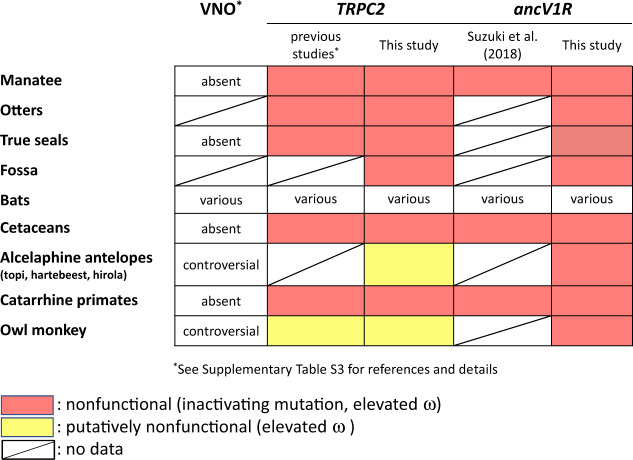
—Summary of the inactivation of *ancV1R* and *TRPC2* sequences identified in the previous studies and this study. The status of the anatomy of the VNO is also shown for comparison. Note that the inactivation of *ancV1R* is correlated with the inactivation of *TRPC2* and with the loss of functional VNO. Alcelaphine antelopes and the owl monkey are indicated as “putatively nonfunctional” in the *TRPC2* column because they are intact at the sequence level but the ω values are elevated to almost 1.0 (see “Results”). The anatomical status for alcelaphine antelopes (topi, hartebeest, and hirola) is also controversial because they possess intact VNO structures but lack flehmen responses. *ancV1R* of true seal in Suzuki et al. (2018) is indicated as “no data” because the ω value was not available. (See supplementary table S3, Supplementary Material online, for more details and references.)

*TRPC2* (transient receptor potential C), which is specifically expressed in most VNO sensory neurons, was shown to be essential for a fully functional VNO (Stowers et al. 2002; Liman and Dulac 2007). Previous studies reported that the inactivation of *TRPC2* is correlated with the loss of anatomically intact VNO in catarrhine primates (Liman and Innan 2003; Zhang and Webb 2003), cetaceans (Yu et al. 2010), and some bats (Zhao et al. 2011; Yohe et al. 2017, 2018), otters, and true seals (Yu et al. 2010, Hecker et al. 2019). Thus, *TRPC2* has been used as a genetic marker to examine the existence of a functional VNO (summarized in fig. 1).

Recently, we characterized a novel VNO-specific gene, named *ancV1R* (Suzuki et al. 2018). *ancV1R* belongs to the vomeronasal receptor type-1 (*V1R*) gene family, which encodes members of a pheromone receptor family and is highly variable in number and repertoire among mammals due to extensive gene gain and loss (Grus et al. 2005; Young et al. 2010, Silva and Antunes 2017; Nikaido 2019). However, *ancV1R* is quite distinct from canonical *V1R*s of mammals in that just one orthologous gene is shared among most bony vertebrates. In addition, *ancV1R* is expressed in all VNO sensory neurons and coexpressed with canonical *V1R*s, implying the essential role of *ancV1R* in the function of the VNO (Suzuki et al. 2018).

In this study, we conducted comprehensive analyses on *ancV1R* sequences using all currently available vertebrate genome assemblies in the NCBI database. The *ancV1R*s were investigated based on two criteria: 1) the presence/absence of inactivating mutation(s), which includes frameshift or premature stop codon mutations; and 2) the ω (d*N*/d*S*) ratios, which can be an indicator of the selective pressure acting on a protein-coding gene. As a result, the inactivating mutations were observed in cetaceans, sirenians, catarrhine primates, and most bat lineages, supporting our previous study (Suzuki et al. 2018, fig. 1). The elevation of ω ratios were also observed in these groups strongly suggesting the relaxation of purifying selection. The relaxation of purifying selection on *ancV1R* is consistent with the loss of functional VNO in these groups. These results demonstrate that the presence/absence of inactivating mutation(s) as well as the ω ratios of *ancV1R* can be used as an ideal genetic marker to evaluate the biological status of the VNO. By applying *ancV1R* as a reliable diagnostic indicator of VNO function, we revealed the relaxation of purifying selection in *ancV1R*s for true seals, the sea otter, the giant otter, the fossa, the owl monkey, and alcelaphine antelopes, in which the existence of a functional VNO is still under debate. To evaluate the possibility of VNO loss in these species, we additionally investigated *TRPC2*s and found the signatures for the relaxation of purifying selection, supporting the results of *ancV1R*. By showing the relaxation of purifying selection in *ancV1R* as well as *TRPC2*, we propose the possibility that the VNOs were degenerated in these species. Thus, *ancV1R* together with additional molecular markers may provide valuable new insights into the neuroanatomical status of the VNO in a broad diversity of mammals.

## Materials and Methods

### Sequence Collection, Alignments, and Inactivating Mutations

Nucleotide tBLASTn searches were performed using intact *ancV1R* sequences identified by a previous study (Suzuki et al. 2018) as queries against all vertebrate species with genomic sequences in the NCBI whole-genome shotgun contig database. A representative *ancV1R* sequence in each order was used as the query for BLAST searching against genomes of all species in the same order. Complete *ancV1R* sequences were identified from 341 species, which include four orders of ancient fishes, two orders of amphibians, four orders of reptiles, and 23 orders of mammals (summarized in supplementary table S1, Supplementary Material online). We excluded the *ancV1R* sequences, which contain multiple “N” base call ambiguities due to reference assembly problems. We also excluded the redundant *ancV1R* sequences of closely related species belonging to the same genus and used only one representative species (see supplementary table S1, Supplementary Material online). In total, 298 sequences were used for the analyses. All obtained nucleotide sequences were translated into amino acid sequences, and the sequences containing premature stop codons were identified as pseudogenes using Biopython (Cock et al. 2009) and visual inspection. The remaining amino acid sequences were identified as intact and aligned in MAFFT (Katoh et al. 2002) using default parameters of mafft-linsi (Katoh et al. 2005). This multiple alignment of amino acid sequences was converted into a codon alignment by PAL2NAL (Suyama et al. 2006). To align nucleotide sequences for pseudogenes into this codon alignment, we used the “–add” option (Katoh and Frith 2012) in MAFFT and minor adjustments by eye (supplementary alignment file 1, Supplementary Material online). We also used this integrated multiple alignment to inspect inactivating mutations, including frameshifts, altered start codons, and premature stop codons. The mutations of inactivated *ancV1R* sequences were inspected to distinguish between two possibilities (assembly error or true inactivation) by megaBLAST searches against the multiple reads of their corresponding assemblies (NCBI short read archive [SRA] data: https://www.ncbi.nlm.nih.gov/sra/, last accessed December 13, 2019). In addition to *ancV1R*, we also mined the longest exon 12 of *TRPC2* (720 bp, starting from phase 0 of the reading frame) for Afrotheria, Primates, Carnivora, Cetartiodactyla, and Chiroptera, using *TRPC2* sequences of cow and mouse (Ensembl ID: ENSBTAE00000396459 and ENSMUSE00001323118) as queries. The resultant sequences were inspected for the presence/absence of inactivating mutations. The entire alignment for the exon 12 of *TRPC2* is shown in supplementary alignment file 2, Supplementary Material online.

### Phylogenetic Analysis

Phylogenetic trees for *ancV1R* genes were generated using RAxML 8.2.4 (Guindon and Gascuel 2003) with the GTR + I + G nucleotide substitution model. The best-fitting nucleotide substitution model was selected by jModelTest2 (Darriba et al. 2012) based on the Akaike information criterion (AIC) scores. Rapid bootstrap analyses were performed with 1,000 replicates for assessing the reliability of nodes. We also used some sequences of fish *V1R5* and *V1R6* included in Suzuki et al. (2018) as outgroups.

### Selection Analyses

Calculations of the nonsynonymous to synonymous ratios (ω  = d*N*/d*S*) for measuring natural selection on sequences of *ancV1R* or exon 12 of *TRPC2* were performed with the codeml program in PAML 4.8 (Yang 2007). Generally, functional genes are under purifying selection and their ω values are <1, but the ω values for nonfunctional genes are elevated to 1. We used the branch models (Yang 1998; Yang and Nielsen 1998) in codeml for examining changes of ω values between foreground and background branches. Selection analyses were performed with separate data sets for each lineage that have inactivated *ancV1R* or *TRPC2* and their corresponding backgrounds which contain all intact sequences in the same order. In the present study, we included the pseudogenized sequences in the selection analyses because the inactivation should be validated from several lines of evidence, 1) existence of the frameshift or premature stop codon, 2) existence of the inactivating mutation in SRA, and 3) elevation of ω value. We used branch-site model in codeml for examining the possibility of positive selection in *ancV1R*s with high ω values without frameshift or premature stop codon mutations. We also used free ratio model in codeml for exploratory search of the branches with elevated ω values of *ancV1R*s in the mammalian tree. For this analysis, we eliminated the *ancV1R* sequences for which the relaxation of purifying selection was examined in detail by using branch model analyses and statistical test.

The data sets were composed of subsets of aligned sequences extracted from the comprehensive sequence alignment (supplementary alignment file 1 or 2, Supplementary Material online) in accordance with the tree topologies for foreground and background branches (fig. 3). Frameshift insertions, lineage-specific in-frame insertions, and stop codons were deleted prior to each selection analysis. For the tree topologies of mammals used in codeml analyses, we referred to the Open Tree of Life database (Hinchliff et al. 2015; supplementary fig. S2, Supplementary Material online). For the tree topology for antelopes, which was not available in the database, we referred to Bärmann et al. (2013) and Chen et al. (2019).

The most appropriate codon frequency models for each selection analysis were selected from the following list: CF0 (codon frequencies are assumed to be equal), CF1 (codon frequencies are calculated from the average nucleotide frequencies), CF2 (codon frequencies are calculated from the average nucleotide frequencies at the three codon positions), and CF3 (codon frequencies are used as free parameters), based on AIC scores of the analyses which performed with all intact *ancV1R* or *TRPC2* sequences of mammals. CF0 was found to be the most appropriate model for *ancV1R* (supplementary table S4, Supplementary Material online) and CF1 was for *TRPC2* (supplementary table S5, Supplementary Material online). To evaluate the significance of ω for foreground branches, likelihood ratio tests were used to compare likelihoods of the multiple ω category models which assumed selective pressures were changed at the foreground branches against that of their corresponding one ω category models which assumed selective pressures were not changed.

## Results

### Characterization of anc*V1R*s in Vertebrates

**Fig. 2.**
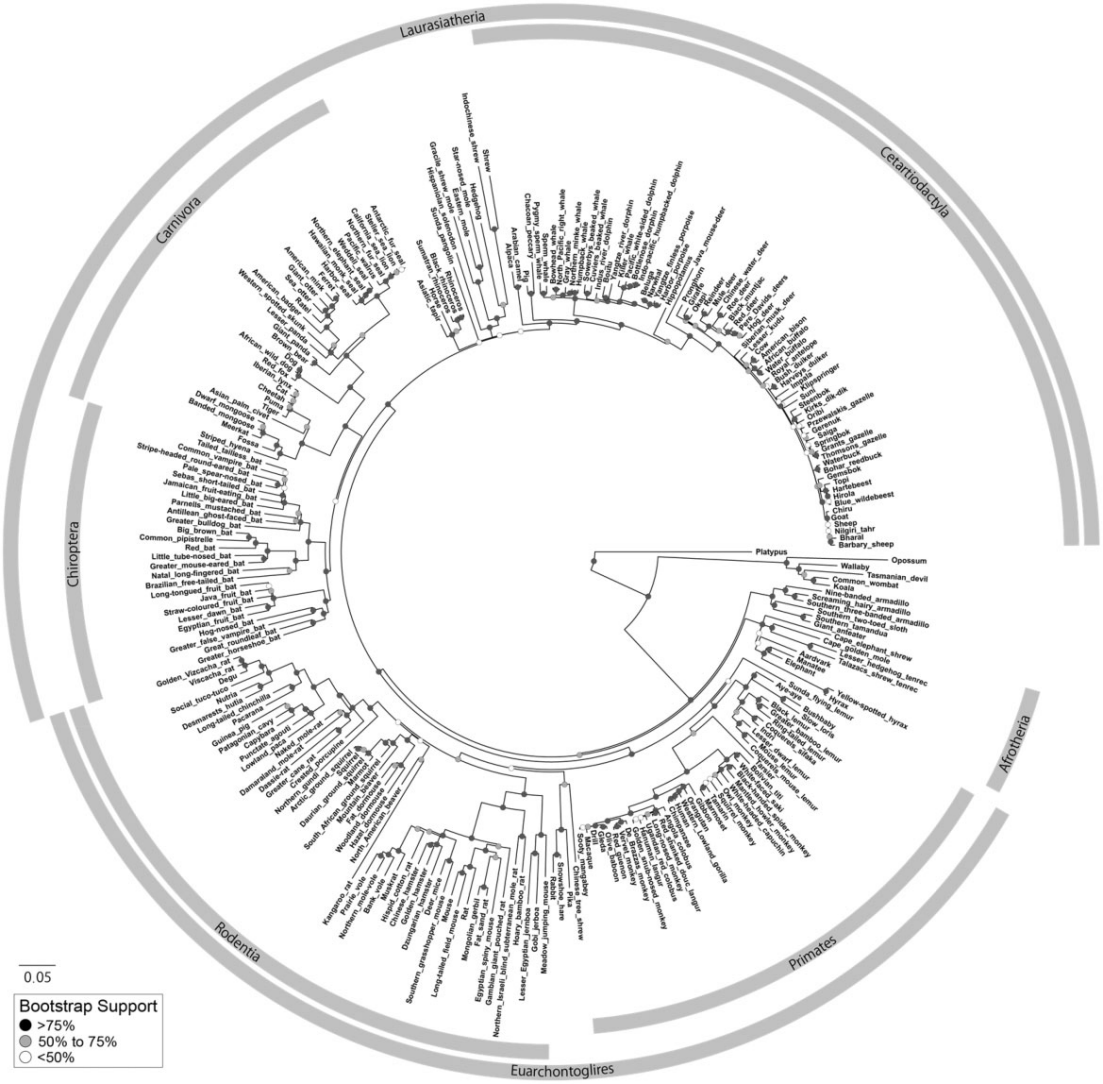
—The maximum likelihood tree of *ancV1R* gene tree for 261 mammals. Gray rectangles indicate members of major orders and superorders. Colored circles on each node indicate bootstrap values described in the legend. Scale bar indicates the number of nucleotide substitutions/site. The phylogenetic tree including all bony vertebrates is shown in the supplementary figure S1, Supplementary Material online.

In the present study, we extensively analyzed a total of 298 *ancV1R* sequences of vertebrates (see details for Materials and Methods). *ancV1R*s were not found in teleosts and birds and were inactivated by many insertions and deletions in crocodilians and testudines (crocodiles, alligators, and turtles; supplementary table S3, Supplementary Material online), which confirm our previous study (Suzuki et al. 2018) and are consistent with the absence of intact VNO in these species (e.g., Silva and Antunes 2017). We found only one copy of *ancV1R* in most species except for the African clawed frog, which has both an intact gene and a pseudogene on chromosomes 1S and 1L, respectively. Importantly, all *ancV1R* sequences identified in this study are located in the first intron of synuclein alpha-interacting protein (*SNCAIP*), suggesting that the synteny relationship between *ancV1R* and *SNCAIP* is conserved among all bony vertebrates from the polypterus to mammals (Suzuki et al. 2018). Supplementary table S2, Supplementary Material online, summarizes the variation in length of the intact *ancV1R*s of mammals. In the intact sequences, the lengths are 972 bp in most cases with some exceptions due to in-frame insertions or deletions. Figure 2 and supplementary figure S1, Supplementary Material online, show the maximum-likelihood tree of *ancV1R* including intact and pseudogene sequences. The teleost *V1R5* and *V1R6* were used as outgroups of *ancV1R* because of their phylogenetic closeness (Suzuki et al. 2018). The resultant *ancV1R* gene tree is mostly consistent with species tree. The two lines of evidence, the conservation of the synteny relationship as well as the consistency in gene tree and species tree, suggest that the *ancV1R* sequences obtained in the present study are single copy and orthologous.

### Relaxation of Purifying Selection in *ancV1R* of the Manatee, Cetaceans, and Catarrhines

Next, we focused on the inactivating mutations of *ancV1R*s in mammals. The inactivation of *ancV1R*s in the manatee, cetaceans, and catarrhines are consistent with the previous study of *ancV1R* (fig. 1; Suzuki et al. 2018). More specifically, the manatee has two frameshift insertions, two nonsense mutations, and one frameshift deletion (fig. 3*A*). Cetaceans share one frameshift deletion that has occurred on the stem cetaceans branch (fig. 3*B*). In catarrhine primates, each of the three families of catarrhines (Hominidae, Cercopithecidae, and Hylobatidae) has independent inactivating mutations, which are not shared among the different families. Hominidae shares one nonsense mutation, Cercopithecidae has one frameshift deletion and one frameshift insertion, and Hylobatidae has one frameshift deletion and one nonsense mutation (fig. 3*C*). These data add further evidence that *ancV1R*s are not intact in the three groups. In addition to the inactivating mutations, we newly estimated selective pressures on each lineage by calculating and comparing the ω (=d*N*/d*S*) ratio. The selection analyses for the manatee, cetaceans, and catarrhines were performed with two ω categories to examine whether or not selective pressures are relaxed in these lineages: 1) a category for each foreground branch for the manatee, cetaceans, or catarrhines and 2) a category for each background branch of afrotherians, cetartiodactyls and perisodactyls, and primates, respectively (fig. 3*A*–*C*). The ω values for the manatee (1.99), cetaceans (1.09), and catarrhines (1.08) are higher than their background ω values (0.38, 0.46, and 0.44), indicating that purifying selection on background branches is relaxed in these lineages, which were all supported by likelihood ratio test (*P *=* *2.7* *×* *10^−4^, 1.4* *×* *10^−7^, and 1.0* *×* *10^−5^; supplementary table S6, Supplementary Material online).

**Fig. 3.**
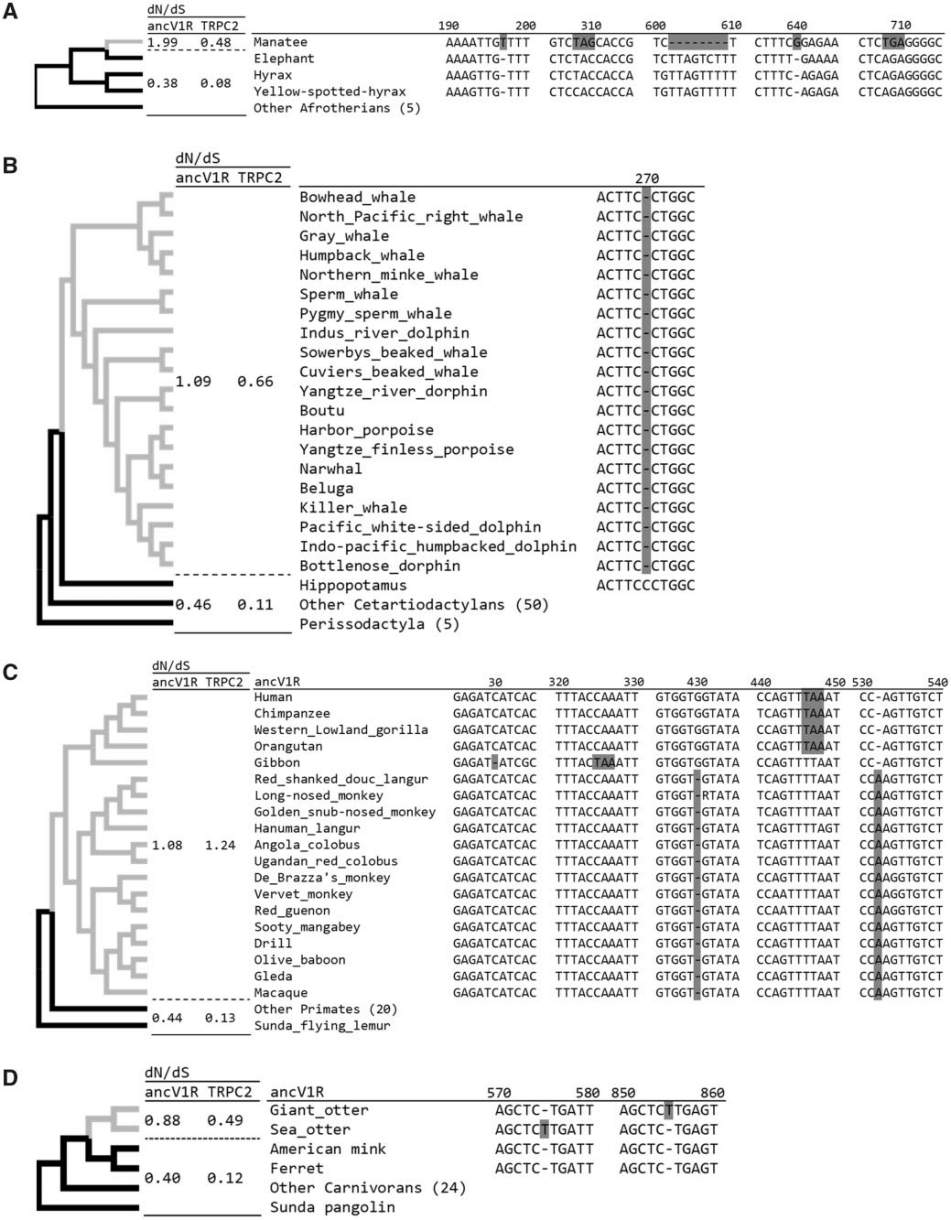
**—**The sequence alignments of *ancV1R* showing the inactivating mutations shared among each family for the manatee (*A*), cetaceans (*B*), catarrhines primates (*C*), otters (*D*), true seals (*E*), the fossa (*F*), the owl monkey (*G*), alcelaphine antelopes (*H*), and bats (*I*), with closely related outgroups. Inactivating mutations are highlighted in gray. Start codon mutations and stop codon mutations are highlighted in yellow and blue, respectively. Each alignment is extracted from the alignment of all *ancV1R* sequences used in this study (supplementary alignment file 1, Supplementary Material online). The numbers above the line indicate the nucleotide positions for each alignment. The colors of each branch indicate different ω categories. The ω values of *ancV1R* and *TRPC2* for each branch are indicated in the d*N*/d*S* columns.

**Fig. 3.**
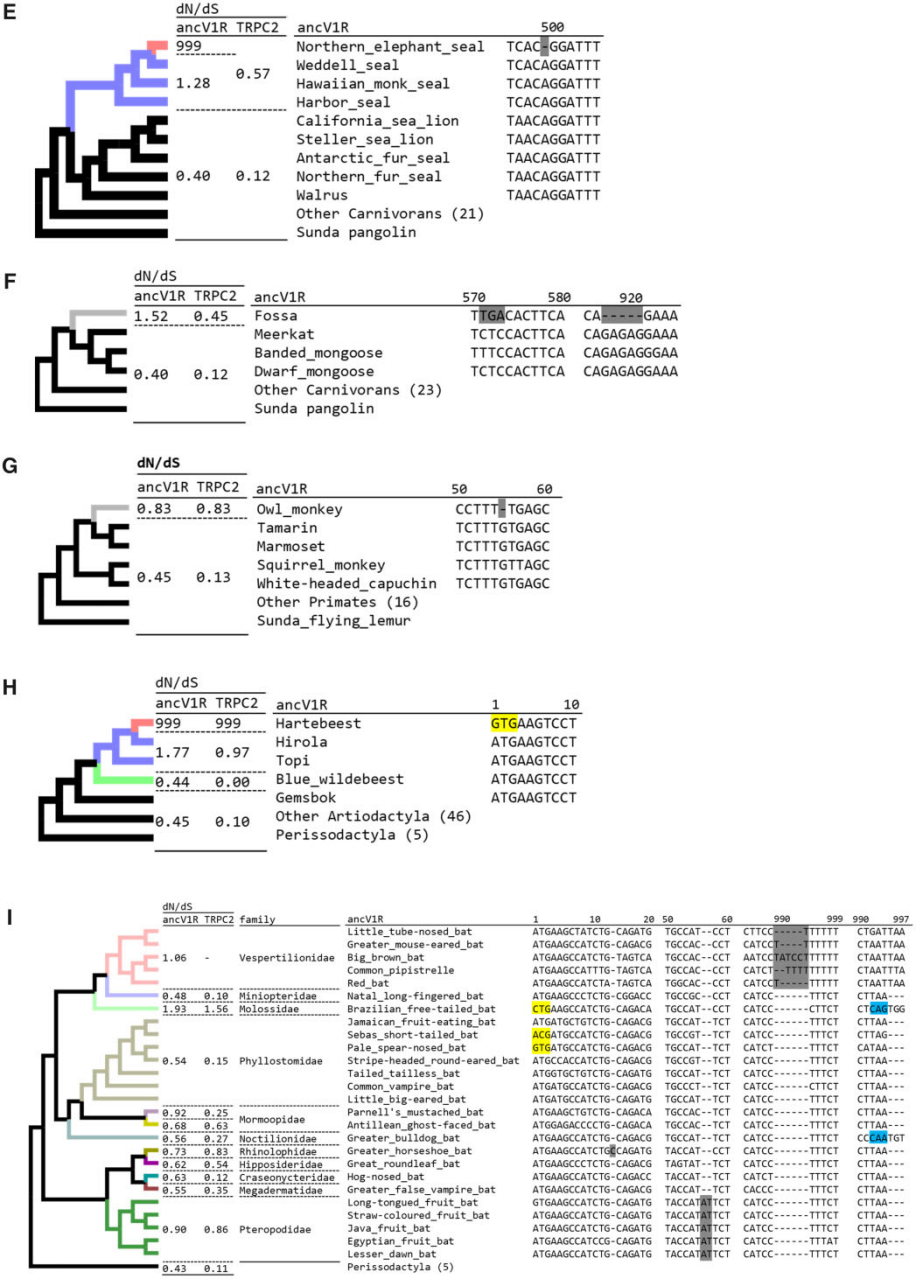
**—**Continued

In the manatee, cetaceans, and catarrhines, we also investigated the exon 12 of *TRPC2* using the same strategies with *ancV1R*. We found inactivating mutations and signatures for the relaxation of purifying selection (supplementary fig. S3*A*–*C*, Supplementary Material online). The ω values for the manatee (0.48), cetaceans (0.66), and catarrhine primates (1.24) are higher than their backgrounds (0.08, 0.11, and 0.13), which were all supported by likelihood ratio test (*P *=* *5.0* *×* *10^−6^, 3.2* *×* *10^−21^, and 1.4* *×* *10^−21^; supplementary table S7, Supplementary Material online). Thus, the signatures for the relaxation of purifying selection on *ancV1R* and *TRPC2* are correlated with each other in these groups. It is noteworthy that the ω values are lower in *TRPC2* than in *ancV1R* for all comparison regardless of the relaxation of purifying selection. The lower ω values of *TRPC2* imply that the selective constraint on amino acid sequence is strict in this protein compared with *ancV1R*.

### Additional Signatures for the Relaxation of Purifying Selection in *ancV1R* of Semiaquatic Mammals

In addition to the previously described species, we newly discovered the inactivations of *ancV1R* in semiaquatic mammals including otters and true seals. We found that *ancV1R*s of both giant otter and sea otter are inactivated by a frameshift mutation at different positions (fig. 3*D*). The ω value of otters (0.88) is higher than background (0.40, *P *=* *0.029; supplementary table S6, Supplementary Material online) and suggests that selective constraints on *ancV1R* were relaxed by aquatic adaptation. We found the signature for relaxation of purifying selection in true seals but not in other pinnipeds (fur seals, walrus). Among the four true seals, *ancV1R* of northern elephant seal is inactivated by a frameshift deletion and the others are intact (fig. 3*E*). To assess whether selective constraints are relaxed not only in northern elephant seal but also in the other three true seals, selection analyses were performed for true seals with three ω categories that separated categories for northern elephant seal, the other three true seals, and background. The elevated ω value of 999 in northern elephant seal is due to the absence of synonymous substitutions. The ω value (1.28) in three true seals is higher than background (0.40) even though that they are intact at the sequence level. These results are supported by the likelihood ratio test (*P *=* *0.0068; supplementary table S6, Supplementary Material online), suggesting that relaxation of purifying selection on *ancV1R*s in all true seals.

We next performed the evolutionary analyses on *TRPC2* of otters and true seals. Although exon 12 of *TRPC2* were intact at the sequence level in giant otter and sea otter, ω value of the otters (0.49) is higher than background (0.12, fig 3*D*;*P *=* *4.2 × 10^−4^, supplementary table S7, Supplementary Material online). The results are consistent with the previous studies showing that river otter has a nonsense mutation (Yu et al. 2010) and sea otter has a mutation at the splicing site (Hecker et al. 2019). These data suggest that selective constraint on *TRPC2*s were relaxed among otters of the subfamily Lutrinae. In true seals, a nonsense mutation on *TRPC2* is shared among all of the four species (supplementary fig. S3*D*, Supplementary Material online). The ω value in true seals (0.57) is higher than background (0.12) (fig. 3*E*;*P *=* *8.7 × 10^−7^, supplementary table S7, Supplementary Material online). Thus, the relaxation of purifying selection on *TRPC2* are consistent with the results of *ancV1R* in otters and true seals.

### Further Signatures for the Relaxation of Purifying Selection in *ancV1R* of Several Terrestrial Mammals

We further discovered the inactivating mutations on *ancV1R* in the fossa, the owl monkey, and alcelaphine antelopes. *ancV1R* of the fossa has one nonsense mutation and one frameshift deletion (fig. 3*F*). *ancV1R* of the owl monkey is also inactivated by one frameshift deletion (fig. 3*G*). Although it is not statistically significant, the ω values of the fossa (1.52) and the owl monkey (0.83) are higher than their background (0.40, 0.40; *P *=* *0.055, 0.26; supplementary table S6, Supplementary Material online). The present findings of the inactivating mutations and elevation of ω values in the fossa and the owl monkey suggest the relaxation of purifying selection on *ancV1R*s in these species.

In the fossa, we revealed that *TRPC2* is inactivated by a nonsense mutation and the ω value (0.45) is higher than background (0.12, supplementary fig. S3*E*, Supplementary Material online; *P *=* *0.0055, supplementary table S7, Supplementary Material online). In the owl monkey, *TRPC2* is intact but the ω value (0.83) is higher than background (0.13) (fig. 3*G*;*P *=* *0.028, supplementary table S7, Supplementary Material online), which is consistent with the previous study (Liman and Innan 2003). Taken together, selective constraints are relaxed in *ancV1R* of the fossa and the owl monkey, which are supported by the results of *TRPC2*.

Among four alcelaphine antelopes (the hartebeest, the hirola, the topi, and the blue wildebeest), *ancV1R*s are intact at the sequence level except for the hartebeest, which has a start codon mutation (fig. 3*H*; in-frame “ATG” was not found within 100 bp upstream or downstream). It is worth noting that flehmen response, which is a typical behavior for catching pheromones, is observed solely in blue wildebeest but not in the hartebeest, the hirola, and the topi (Hart et al. 1988). To examine whether or not selective pressure was relaxed in alcelaphine antelopes, we assigned separate ω categories for branches of the hartebeest, the blue wildebeest, and the hirola and the topi. The ω value of 999 for the hartebeest branch is due to the absence of synonymous substitutions. The ω value for hirola and topi (1.77) is higher than background (0.45, fig. 3*H*). The ω value for the blue wildebeest (0.44) is similar to background (0.45). Although the result is not significant by likelihood ratio test (*P *=* *0.091, supplementary tabel S6, Supplementary Material online), the selective constraint on *ancV1R* is likely to be relaxed in the hartebeest, the hirola, and the topi but not in the blue wildebeest.

In alcelaphine antelopes, exon 12 of *TRPC2* are all intact at the sequence level. However, the ω values of the hartebeest (999, absence of synonymous substitutions), the hirola and the topi (0.97) are higher than background (0.10). The ω value of blue wildebeest (0.00, absence of nonsynonymous substitutions) was similar to background (0.10). This result is significant by likelihood ratio test (fig. 3*H*;*P *=* *0.001, supplementary table S7, Supplementary Material online). Thus, the *TRPC2* show same evolutionary trend with *ancV1R* in that the selective constraint was relaxed in the hartebeest, the hilora, and the topi but not in the blue wildebeest. The results of *ancV1R* and *TRPC2* are both consistent with behavioral study in that the flehmen response is observed solely in the blue wildebeest among alcelaphine antelopes.

### Existence of Intact and Inactivated *ancV1R*s in Bats

Previously, Suzuki et al. (2018) analyzed eight bat species and identified the inactivation of *ancV1R*s in three (Vespertilionidae, Rhinolophidae, and Pteropodidae) of five families. In the present study, we newly added a total of 11 families (33 species) for the analyses. Inactivation of the VNO, *ancV1R*, and *TRPC2* were correlated in most bats but not in some bats (supplementary table S8, Supplementary Material online). The precise sequence alignment revealed that *ancV1R*s are inactivated in four (Vespertilionidae, Rhinolophidae, Molossidae, and Pteropodidae) of 11 families (fig. 3*I*). The frameshift insertions independently occurred in Pteropodidae (long-tongued fruit bat, etc.) and Rhinolophidae (greater horseshoe bat). Vespertilionidae (little tube-nosed bat, etc.) possess a frameshift insertion except for the big brown bat, in which a 6-bp insertion at the same site makes *ancV1R* apparently in-frame (fig. 3*I*). In Molossidae (Brazilian free-tailed bat), start and stop codon positions are both moved to 16 and 25 bp downstream, respectively, which may inactivate *ancV1R*.

Next, we estimated the ω values of *ancV1R* and *TRPC2* for each family of bats. Given that there are several bat families which have intact *ancV1R* in spite that their VNO and *TRPC2* are inactivated (e.g., Mormoopidae, Noctilionidae, Hipposideridae, Megadermatidae; supplementary table S8, Supplementary Material online), we assigned separate ω categories to each family to evaluate ω values. The ω values of *ancV1R* for Pteropodidae (0.90), Rhinolophidae (0.73), Molossidae (1.93), and Vespertilionidae (1.06) are higher than background (0.43) (fig. 3*I*;*P *=* *4.8 × 10^−4^, supplementar table S6, Supplementary Material online), suggesting the relaxation of purifying selection in these families. The ω values of *TRPC2* for Pteropodidae (0.86), Rhinolophidae (0.83), and Molossidae (1.56) are higher than background (0.11) (fig. 3*I*;*P *=* *2.3 × 10^−13^, supplementary table S7, Supplementary Material online), except for Vespertilionidae, in which exon 12 of *TRPC2* were not found in the genome. The results of *ancV1R* and *TRPC2* are both consistent with the absence of intact VNOs in these four families.

The ω values of *ancV1R* for Phyllostomidae (Jamaican fruit-eating bat, etc.) (0.54) and Miniopteridae (Natal long-fingered bat) (0.48) were similar to the background (0.43). The ω values of *TRPC2* for Phyllostomidae (0.15) and Miniopteridae (0.10) were also similar to background (0.11) (fig. 3*I*). The results of *ancV1R* and *TRPC2* are both consistent with that they possess intact VNOs (Yohe et al. 2017).

The inactivation of *ancV1R* and the loss of an anatomically intact VNO as well as the inactivation of *TRPC2* are tightly correlated in most bat species analyzed. However, such correlations were somewhat weak in the following bat families, Hipposideridae (great roundleaf bat), Megadermatidae (greater false vampire bat), and Noctilionidae (greater bulldog bat). Although they possess intact *ancV1R*s, of which the ω values (fig. 3*I*; 0.55–0.63) are slightly higher than bats with intact VNOs (0.48–0.54), their VNOs are vestigial and *TRPC2*s are inactivated (supplementary table S8, Supplementary Material online). It is difficult to conclude whether or not purifying selection was relaxed on *ancV1R*s in these groups. Furthermore, in Mormoopidae, the ω values of intact *ancV1R* in Parnell’s mustached bat (0.92) and in Antillean ghost-faced (0.68) are not consistent with the presence/absence of intact VNO (supplementary table S8, Supplementary Material online). We performed additional analyses using branch-site model in codeml and found no signatures for positive selection (supplementary table S9, Supplementary Material online).

### Examples of Ambiguous Inactivation

The southern two-toed sloth has a 4-bp deletion in its sequence (supplementary fig. S4, Supplementary Material online), which was confirmed in multiple reads (SRX4501348). However, the ω value for this species (0.30) is lower than backgrounds (0.40). In addition, *TRPC2* is intact and the ω value (0.08) is also lower than background (0.15) (supplementary fig. S4, Supplementary Material online). We also found that the closely related species of three-toed sloth has intact *ancV1R*. Thus, it is possible that the 4-bp deletion exists only in the sequenced individual and/or was very recent evolutionary event. To examine these possibilities, we need a polymerase chain reaction validation of the 4-bp deletion for multiple individuals of wild populations in future study.

### Exploratory Analyses for the Elevation of ω Values in *ancV1R*s

In addition to the hypothesis testing method using branch model, we also comprehensively explored the branches with elevated ω values of *ancV1R*s in mammalian tree by using free ratio model. Supplementary figure S5, Supplementary Material online, showed the summary of ω values on each branch. As a result, we found that the ω values are around 0.4 in most of the branches, suggesting the operation of purifying selection in these mammals analyzed. However, we also detected apparent elevation of ω values in several species (ω > 0.8; supplementary table S10A, Supplementary Material online), which were subjected to the additional analyses using branch model and likelihood ratio test. As a result, the elevation of ω values were statistically significant in lesser panda (*P *=* *0.043) and Pacarana (*P *=* *0.0064). The branch-site analysis for these species showed that the operation of positive selection in these species is unlikely (supplementary table S10B, Supplementary Material online). To argue the possibility of the degeneration of the VNO, we further examined the *TRPC2*, revealing that the ω values of lesser panda (0.074) and Pacarana (0.070) were comparable with the back ground (0.12 and 0.050, respectively). Thus, ω values were elevated in *ancV1R* but not in *TRPC2.* The results imply that the elevation of ω values in *ancV1R* could be the noise in the low number of mutations, or the selective constraint was relaxed only in *ancV1R* of these species. Given that the description of the VNO still remains limited in lesser panda and Pacarana, these groups could be the next target of the anatomical and molecular investigation.

## Discussion

Understanding the source of phenotypic diversity at the DNA level is a key challenge in molecular evolutionary biology. The coincidental loss of a particular gene and a specific trait may provide important insight into the molecular mechanisms that underlie phenotypic diversity (Sharma et al. 2018). Recently, such phenotype–genotype relationships have been uncovered in several studies of mammals; the inactivation of C4orf26, MC5R, and OMP showed strong correlation with the loss of teeth (Springer et al. 2016), the sebaceous gland (Springer and Gatesy 2018), and olfactory sense (Springer and Gatesy 2017), respectively. Given that *ancV1R* is expressed in all VNO sensory neurons and coexpressed with canonical *V1R*s (Suzuki et al. 2018), we suppose that *ancV1R* possesses general and essential function in the VNO. Therefore, it is of primary interest to examine the correlation between phenotype (VNO) and genotype (*ancV1R*). Here, we found clear correlations between the loss of the VNO and the inactivation of *ancV1R*. Furthermore, we revealed unexpected inactivation of *ancV1R* in multiple lineages of mammals for which the existence of a functional VNO is not well described.

### Consistent Pattern of Inactivation of *ancV1R*, *TRPC2*, and the Loss of the VNO

In all catarrhine primates, our observations of gene inactivation and the elevation of ω values in *ancV1R* and *TRPC2* are consistent with previous studies (Liman and Innan 2003; Zhang and Webb 2003) and anatomical loss of both the VNO and the distinct accessory olfactory bulb (Wysocki 1979; Bhatnagar and Meisami 1998; Smith et al. 2014). The degeneration of the VNO in these groups could be based on an evolutionary trade-off shifting toward more reliance upon visual and auditory signals rather than the chemical signals for reproductive communication.

In cetaceans, the inactivation and the elevation of ω values in *ancV1R* occurred in their stem lineage (fig. 3*B*). This is consistent with the inactivation of *TRPC2* (supplementary fig. S3*B*, Supplementary Material online; Yu et al. 2010) and the absence of the VNO and the accessory olfactory bulb (Lowell and Flanigan 1980; Mackay-Sim et al. 1985). It is noteworthy that the timing of the inactivation of *ancV1R* and *TRPC2* occurred earlier than that of *OMP*, which was inactivated in most toothed whales but is still intact in baleen whales (Kishida et al. 2015; Springer and Gatesy 2017). Springer and Gatesy (2017) noted that the olfactory sense was retained in the common ancestor of Cetacea but was significantly reduced in odontocetes by a trade-off with the evolution of echolocation ability. Given that *ancV1R* was inactivated in both toothed and baleen whales, the vomeronasal system may have been less important than olfaction during the adaptation toward a fully aquatic environment.

In bats, the inactivation and the elevation of ω values in *ancV1R* occurred in Pteropodidae, Rhinolophidae, Vespertilionidae, and Molossidae, which are consistent with the inactivation of *TRPC2* and loss of the VNO (fig. 3*I*; Zhao et al. 2011; Yohe et al. 2017). Conversely, the existence of an intact *ancV1R* and similar ω values with background in Phyllostomidae and Miniopteridae are also consistent with the presence of an anatomically intact VNO in representative species belonging to these two families (Bhatnagar and Meisami 1998). Given that the first inactivating mutations are different among each family (fig. 3*I*), the timing of inactivation of *ancV1R* does not date back to the common ancestor of all bats but rather to the ancestors of each family. These results imply that the loss of the VNO occurred relatively recently and independently in bat evolution, which is also consistent with the finding of Yohe et al. (2017).

### Complex History of the Vomeronasal Sensory System in Bats

Although we obtained clear correlations between phenotype and genotype, we also obtained a few ambiguous results in several bat species investigated. This may reflect the rapid and complex evolutionary history of the vomeronasal sensory system in this group (e.g., Yohe et al. 2017). First, Hipposideridae, Megadermatidae, Noctilionidae, and one species of Mormoopidae (antillean ghost-faced bat) possess intact *ancV1R*s, of which the ω values were only slightly higher than background (fig. 3*I*; 0.62, 0.55, 0.56, and 0.68, respectively). It is difficult to judge whether or not selection pressure on VNO functionality was relaxed by solely focusing on *ancV1R*. However, considering that *TRPC2* was inactivated in these species (Yohe et al. 2017), the slightly higher ω values of *ancV1R* may reflect some relaxation of purifying selection, which is consistent with anatomical observations of lacking VNO (Wible and Bhatnagar 1996). The recent loss of the vomeronasal sensory system in bats may not have allowed sufficient time for the accumulation of deleterious mutations in *ancV1R*, making it difficult to detect an obvious signature for the relaxation of purifying selection. Conversely, Parnell’s mustached bat (Mormoopidae) possesses an intact *ancV1R*, of which the ω value (0.92) was higher than background. The operation of positive selection in this bat species was shown to be unlikely by branch-site test (supplementary table S9, Supplementary Material online), implying the possibility of the relaxation of purifying selection. However, its *TRPC2* is intact (Yohe et al. 2017) and the VNO is also anatomically intact in this species (Bhatnagar and Meisami 1998). These data imply that only *ancV1R* sequence may not be sufficient to judge the presence/absence of functional VNO in this species. To further broaden our understanding about the evolution of the VNO in this complex bat family, investigation of additional *ancV1R* as well as *TRPC2* of closely related species are indispensable. It is also noteworthy that hog-nosed bat (Craseonycteridae) possesses intact *ancV1R* and *TRPC2* with mostly similar ω values with each background (fig. 3*I*). This suggests that VNS is still functional in Craseonycteridae, in spite that most of yinpterochiropteran bats (from Rhinolophidae to Pteropodidae, fig. 3*I*) lack intact VNOs (supplementary table S8, Supplementary Material online).

### Novel Insights into VNO Functionality from *ancV1R* Evolution

By applying *ancV1R* as a reliable and useful genetic marker, we obtained several novel insights into the vomeronasal sensory systems in mammals that was supported by the additional analyses of *TRPC2*. In Pinnipedia, *ancV1R* was inactivated in only one species of true seals (Northern elephant seal). In addition, the elevation of ω values was also observed in three other species of true seals (Weddell seal, Hawaiian monk seal, and harbor seal), implying the relaxation of purifying selection (fig. 3*E*), that was supported by the inactivation of *TRPC2* (supplementary fig. S3*D*, Supplementary Material online; Yu et al. 2010). Thus, both of the two independent molecular markers suggest that the vomeronasal sensory system became nonfunctional in true seals. It is noteworthy that in pinnipeds the existence of the accessory olfactory bulb could not be clearly identified only in true seals investigated (harbor seal; Meisami and Bhatnagar 1998), implying a reduction or loss of vomeronasal sensory system function in true seals. Molecular, morphological, and ecological studies now consistently suggest that true seals are more aquatic than other pinnipeds (Jefferson et al. 2008) and thus are expected to be less dependent on vomero-olfaction.

In the sea otter and the giant otter, *ancV1R*s are inactivated by a frameshift insertion of T at different sites (fig. 3*D*), suggesting that *ancV1R*s were independently inactivated in these species. To our knowledge, the VNO has not been described in otters. Given that the selective constraint on *TRPC2* was also relaxed the river otter (Yu et al. 2010), sea otter, and the giant otter (fig. 3*D*), it is likely that the vomeronasal sensory system became vestigial in the common ancestor of these two groups coinciding with adaptation to aquatic environments. Although a behavioral study described the flehmen response in sea otter, they also pointed out that the response was not stereotypical for this behavior (Island et al. 2017). It is possible that the flehmen response in sea otter could be induced by MOE rather than VNO. It is worth noting that the number of olfactory receptor genes has decreased in both the sea otter and the giant otter (Beichman et al. 2019), implying that aquatic adaptation may have affected not only the vomeronasal but also the olfactory sensory system during the evolution of these species.

We further obtained an unexpected example of inactivation, namely, the frameshift mutation, as well as an elevated ω value (fig. 3*G*) for *ancV1R* in the owl monkey (*Aotus nancymaae*). In a previous study, Liman and Innan (2003) revealed an elevated d*N*/d*S* ratio for *TRPC2* in the owl monkey that was confirmed by our study (fig. 3*C*), suggest that the VNO is either already nonfunctional or else on the way to becoming nonfunctional. Although the possibility of a reduction of the VNO in New World monkeys has been discussed in both anatomical (Smith et al. 2011) and molecular (Liman and Innan 2003; Yoder et al. 2014; Moriya-Ito et al. 2018) studies, no precise description was done in the owl monkeys.

We also found an unexpected example of inactivating mutations as well as an elevated ω value (fig. 3*F*) for *ancV1R* in one species of Malagasy carnivorans; the fossa (*Cryptoprocta ferox*), that is consistent with the existence of premature stop codon in *TRPC2* (supplementary fig. S3*E*, Supplementary Material online). The fossa has once been classified in Viverridae (most civets and their relatives), but it was revealed to be more closely related to Herpestidae (mongooses) and included in the monophyletic group of Malagasy carnivorans (Yoder et al. 2003). To our knowledge, the vomeronasal systems of the fossa and the other carnivorans in Madagascar have not yet been described. Thus, the anatomical status of the VNO and the reproductive behavior of these species represent an important next target of continuing research. It is important to note that an olfactory receptor gene with premature stop codon was shown to encode functional protein due to the efficient translational read-through (pseudo–pseudo gene; Prieto-Godino et al. 2016). Therefore, we cannot completely exclude the possibility that the *TRPC2* are still functional in this species. More comprehensive analyses on VNO-related genes may provide insights into the status of the vomeronasal system in this species.

The relaxation of purifying selection for *ancV1R* in the hartebeest, the hirola, and the topi, and the operation of purifying selection in the blue wildebeest (fig. 3*H*) are all highly consistent with the presence/absence of flehmen responses in alcelaphine antelopes (Hart et al. 1988), which are also supported by *TRPC2*. Anatomically, however, VNO structures were described in all alcelaphine antelopes (Hart et al. 1988). It is important to note that incisive papilla and incisive ducts, which are involved in transferring pheromones from the oral cavity to the VNO, were degenerated in the hartebeest, the hirola and the topi. On the other hand, very small incisive duct still exists in the wildebeest (*Connochaetes taurinus*), implying that the vomeronasal system is retained to be functional in this species. Our analyses for *ancV1R* of alcelaphine antelopes suggest that purifying selection was relaxed in the common ancestor of the topi, the hirola, and the hartebeest, and likely corresponds with the loss of the flehmen response and the complete loss of incisive papilla and ducts in this group. Our study provides the first genetic insight into the evolution of vomeronasal systems in alcelaphine antelopes.

In the present study, our evolutionary analyses of nearly all 23 mammalian orders showed obvious correlations between the phenotype of the VNO and the genotype of *ancV1R*, demonstrating that the presence/absence of inactivating mutation(s) and the ω values of *ancV1R* can be used as valuable genetic markers to elucidate the biological status of the VNO. Furthermore, five unexpected examples of inactivation events in true seals, otters, the owl monkey, the fossa, and alcelaphine antelopes, as revealed by *ancV1R* and *TRPC2* sequences, provide a timely and important opportunity to reexamine the anatomical condition of the VNO in these ecologically diverse species for which VNO functionality remains uncertain.

## Supplementary Material

evaa082_Supplementary_Data

## Notes

**Associate editor:** Yoshihito T. E. Niimura
