# Supplementary material for: Inactivation of ancV1R as a Predictive Signature for the Loss of Vomeronasal System in Mammals
Source: Genome Biol Evol. 2020 Apr 21;12(6):766–78. doi: 10.1093/gbe/evaa082 (PMC7290294; doi:10.1093/gbe/evaa082)
Supplement: evaa082_Supplementary_Data [file evaa082_supplementary_data.zip › Supplementary Table S2-S10.pptx]

## Slide 1
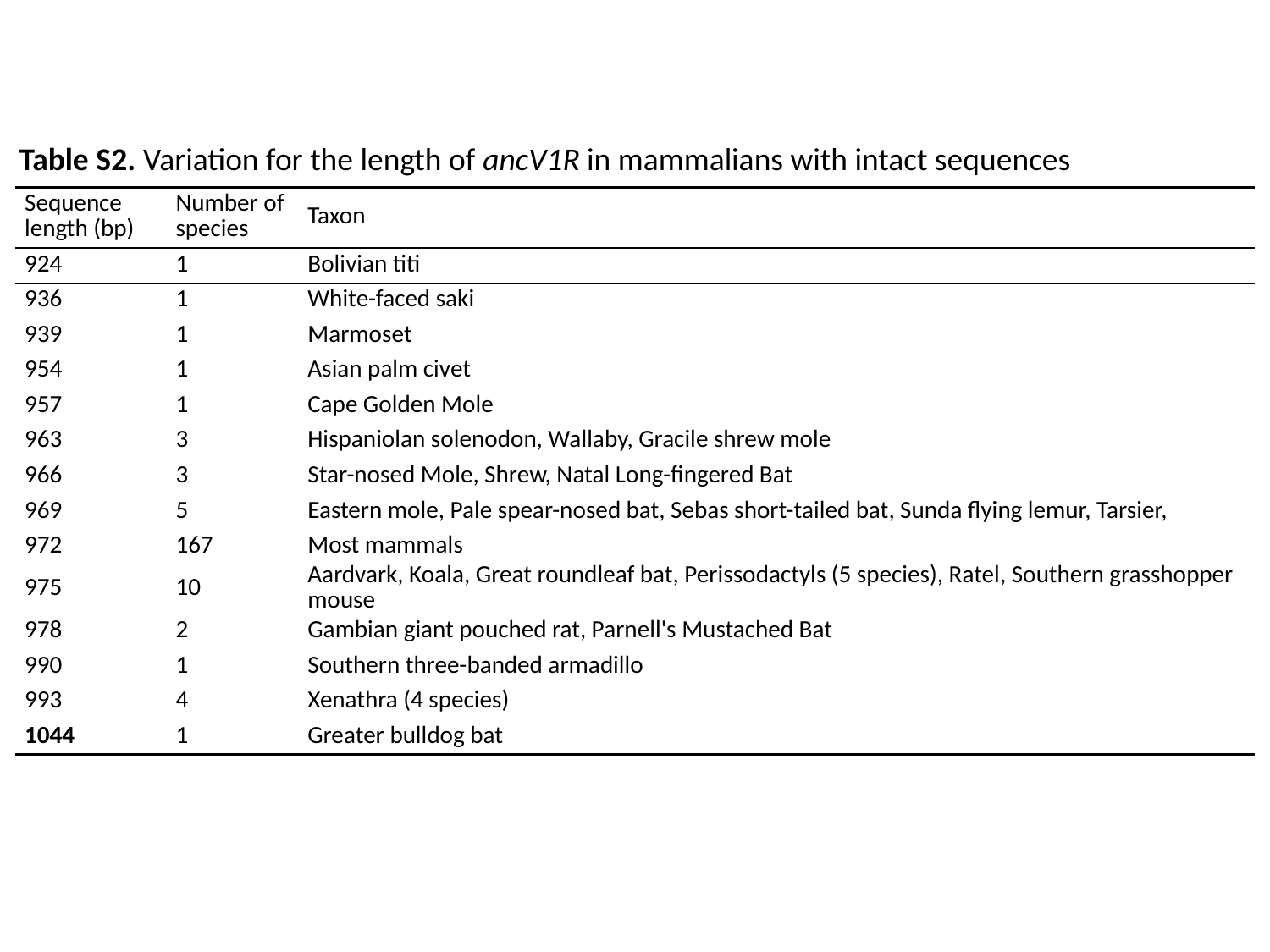

Table S2. Variation for the length of ancV1R in mammalians with intact sequences
| Sequence length (bp) | Number of species | Taxon |
| --- | --- | --- |
| 924 | 1 | Bolivian titi |
| 936 | 1 | White-faced saki |
| 939 | 1 | Marmoset |
| 954 | 1 | Asian palm civet |
| 957 | 1 | Cape Golden Mole |
| 963 | 3 | Hispaniolan solenodon, Wallaby, Gracile shrew mole |
| 966 | 3 | Star-nosed Mole, Shrew, Natal Long-fingered Bat |
| 969 | 5 | Eastern mole, Pale spear-nosed bat, Sebas short-tailed bat, Sunda flying lemur, Tarsier, |
| 972 | 167 | Most mammals |
| 975 | 10 | Aardvark, Koala, Great roundleaf bat, Perissodactyls (5 species), Ratel, Southern grasshopper mouse |
| 978 | 2 | Gambian giant pouched rat, Parnell's Mustached Bat |
| 990 | 1 | Southern three-banded armadillo |
| 993 | 4 | Xenathra (4 species) |
| 1044 | 1 | Greater bulldog bat |

## Slide 2
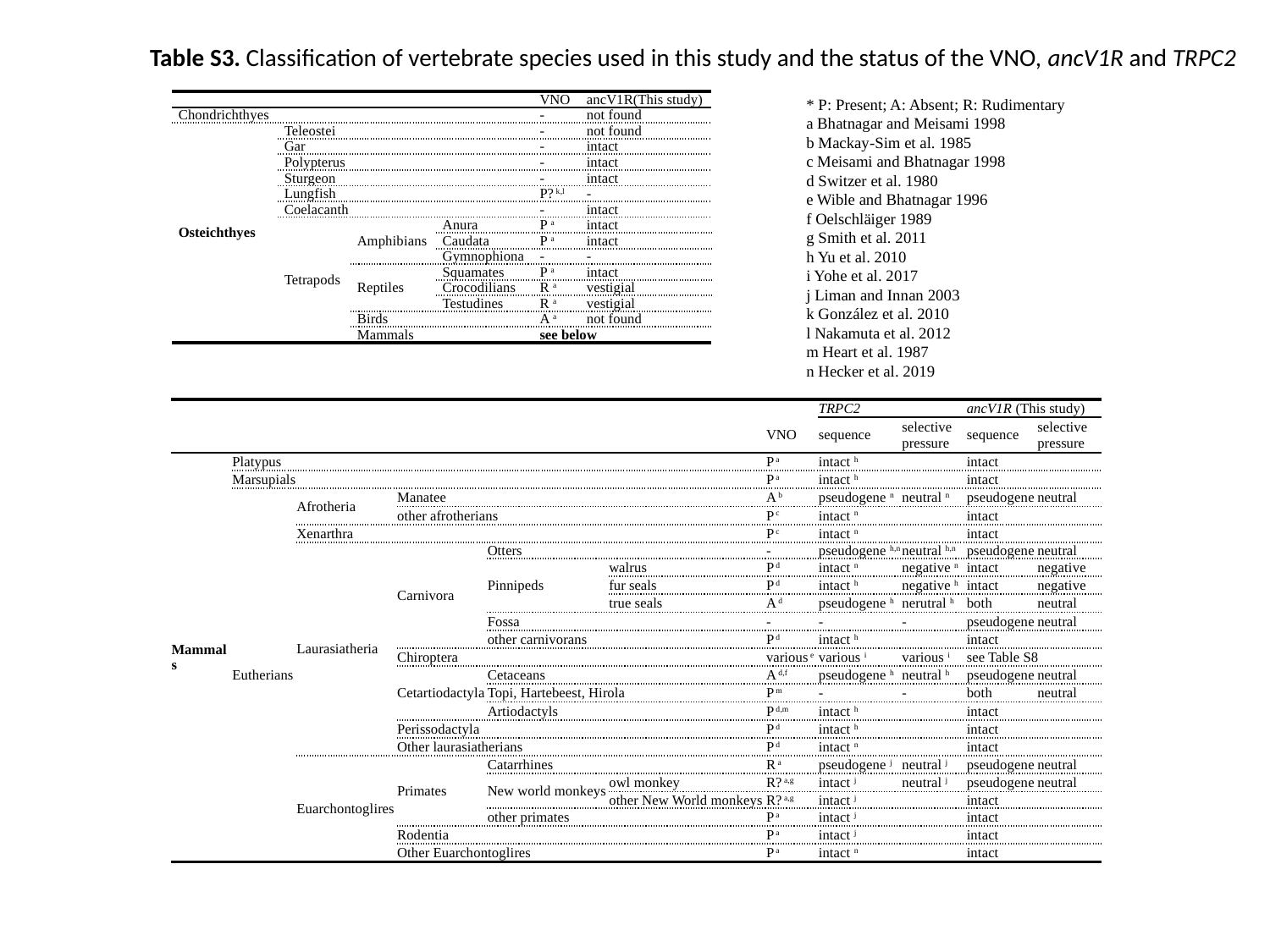

Table S3. Classification of vertebrate species used in this study and the status of the VNO, ancV1R and TRPC2
* P: Present; A: Absent; R: Rudimentary
a Bhatnagar and Meisami 1998
b Mackay-Sim et al. 1985
c Meisami and Bhatnagar 1998
d Switzer et al. 1980
e Wible and Bhatnagar 1996
f Oelschläiger 1989
g Smith et al. 2011
h Yu et al. 2010
i Yohe et al. 2017
j Liman and Innan 2003
k González et al. 2010
l Nakamuta et al. 2012
m Heart et al. 1987
n Hecker et al. 2019
| | | | | VNO | ancV1R(This study) |
| --- | --- | --- | --- | --- | --- |
| Chondrichthyes | | | | - | not found |
| Osteichthyes | Teleostei | | | - | not found |
| | Gar | | | - | intact |
| | Polypterus | | | - | intact |
| | Sturgeon | | | - | intact |
| | Lungfish | | | P? k,l | - |
| | Coelacanth | | | - | intact |
| | Tetrapods | Amphibians | Anura | P a | intact |
| | | | Caudata | P a | intact |
| | | | Gymnophiona | - | - |
| | | Reptiles | Squamates | P a | intact |
| | | | Crocodilians | R a | vestigial |
| | | | Testudines | R a | vestigial |
| | | Birds | | A a | not found |
| | | Mammals | | see below | |
| | | | | | | | TRPC2 | | ancV1R (This study) | |
| --- | --- | --- | --- | --- | --- | --- | --- | --- | --- | --- |
| | | | | | | VNO | sequence | selective pressure | sequence | selective pressure |
| Mammals | Platypus | | | | | P a | intact h | | intact | |
| | Marsupials | | | | | P a | intact h | | intact | |
| | Eutherians | Afrotheria | Manatee | | | A b | pseudogene n | neutral n | pseudogene | neutral |
| | | | other afrotherians | | | P c | intact n | | intact | |
| | | Xenarthra | | | | P c | intact n | | intact | |
| | | Laurasiatheria | Carnivora | Otters | | - | pseudogene h,n | neutral h,n | pseudogene | neutral |
| | | | | Pinnipeds | walrus | P d | intact n | negative n | intact | negative |
| | | | | | fur seals | P d | intact h | negative h | intact | negative |
| | | | | | true seals | A d | pseudogene h | nerutral h | both | neutral |
| | | | | Fossa | | - | - | - | pseudogene | neutral |
| | | | | other carnivorans | | P d | intact h | | intact | |
| | | | Chiroptera | | | various e | various i | various i | see Table S8 | |
| | | | Cetartiodactyla | Cetaceans | | A d,f | pseudogene h | neutral h | pseudogene | neutral |
| | | | | Topi, Hartebeest, Hirola | | P m | - | - | both | neutral |
| | | | | Artiodactyls | | P d,m | intact h | | intact | |
| | | | Perissodactyla | | | P d | intact h | | intact | |
| | | | Other laurasiatherians | | | P d | intact n | | intact | |
| | | Euarchontoglires | Primates | Catarrhines | | R a | pseudogene j | neutral j | pseudogene | neutral |
| | | | | New world monkeys | owl monkey | R? a,g | intact j | neutral j | pseudogene | neutral |
| | | | | | other New World monkeys | R? a,g | intact j | | intact | |
| | | | | other primates | | P a | intact j | | intact | |
| | | | Rodentia | | | P a | intact j | | intact | |
| | | | Other Euarchontoglires | | | P a | intact n | | intact | |

## Slide 3
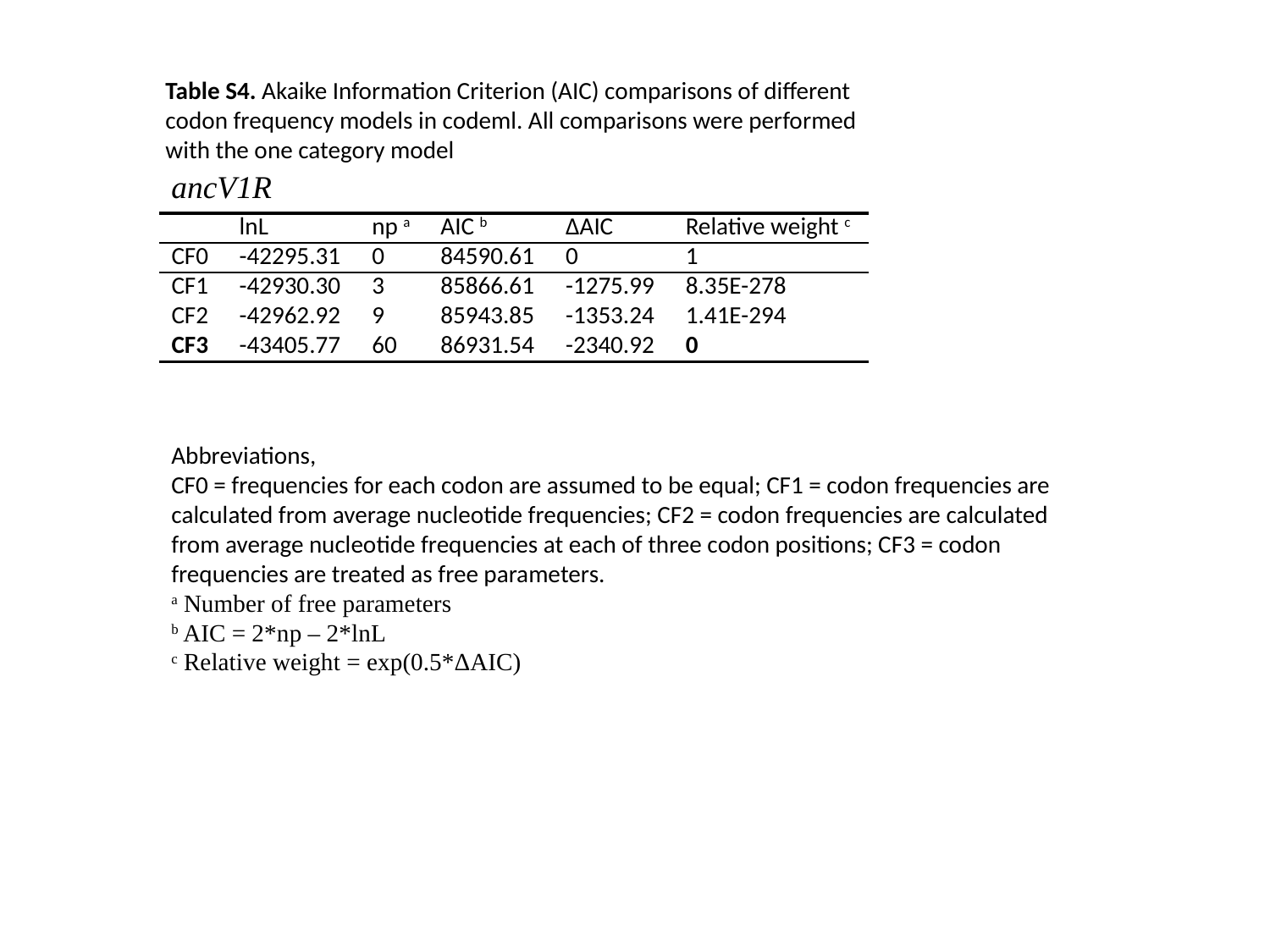

Table S4. Akaike Information Criterion (AIC) comparisons of different codon frequency models in codeml. All comparisons were performed with the one category model
ancV1R
| | lnL | np a | AIC b | ΔAIC | Relative weight c |
| --- | --- | --- | --- | --- | --- |
| CF0 | -42295.31 | 0 | 84590.61 | 0 | 1 |
| CF1 | -42930.30 | 3 | 85866.61 | -1275.99 | 8.35E-278 |
| CF2 | -42962.92 | 9 | 85943.85 | -1353.24 | 1.41E-294 |
| CF3 | -43405.77 | 60 | 86931.54 | -2340.92 | 0 |
Abbreviations,
CF0 = frequencies for each codon are assumed to be equal; CF1 = codon frequencies are calculated from average nucleotide frequencies; CF2 = codon frequencies are calculated from average nucleotide frequencies at each of three codon positions; CF3 = codon frequencies are treated as free parameters.
a Number of free parameters
b AIC = 2*np – 2*lnL
c Relative weight = exp(0.5*ΔAIC)

## Slide 4
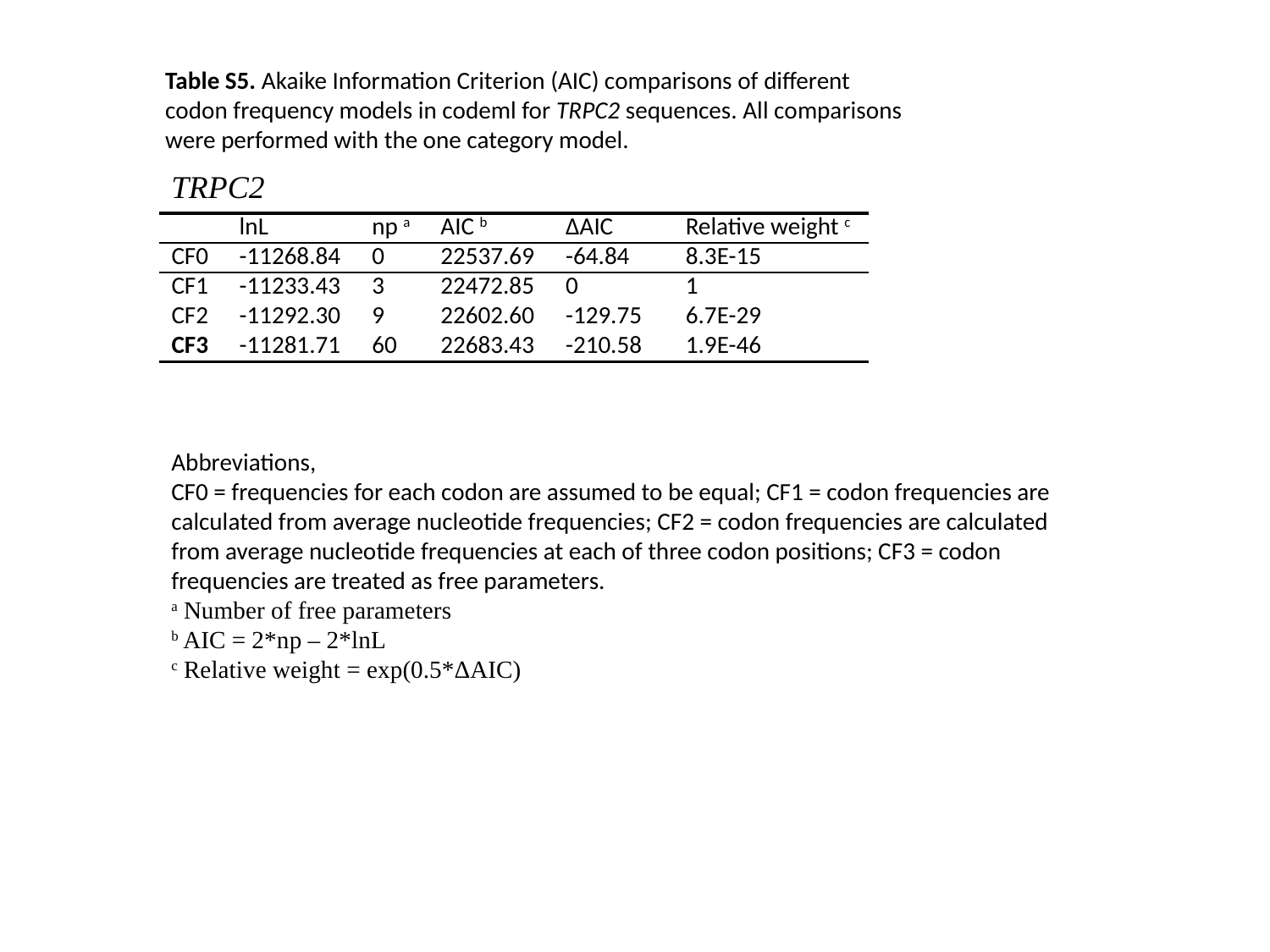

Table S5. Akaike Information Criterion (AIC) comparisons of different codon frequency models in codeml for TRPC2 sequences. All comparisons were performed with the one category model.
TRPC2
| | lnL | np a | AIC b | ΔAIC | Relative weight c |
| --- | --- | --- | --- | --- | --- |
| CF0 | -11268.84 | 0 | 22537.69 | -64.84 | 8.3E-15 |
| CF1 | -11233.43 | 3 | 22472.85 | 0 | 1 |
| CF2 | -11292.30 | 9 | 22602.60 | -129.75 | 6.7E-29 |
| CF3 | -11281.71 | 60 | 22683.43 | -210.58 | 1.9E-46 |
Abbreviations,
CF0 = frequencies for each codon are assumed to be equal; CF1 = codon frequencies are calculated from average nucleotide frequencies; CF2 = codon frequencies are calculated from average nucleotide frequencies at each of three codon positions; CF3 = codon frequencies are treated as free parameters.
a Number of free parameters
b AIC = 2*np – 2*lnL
c Relative weight = exp(0.5*ΔAIC)

## Slide 5
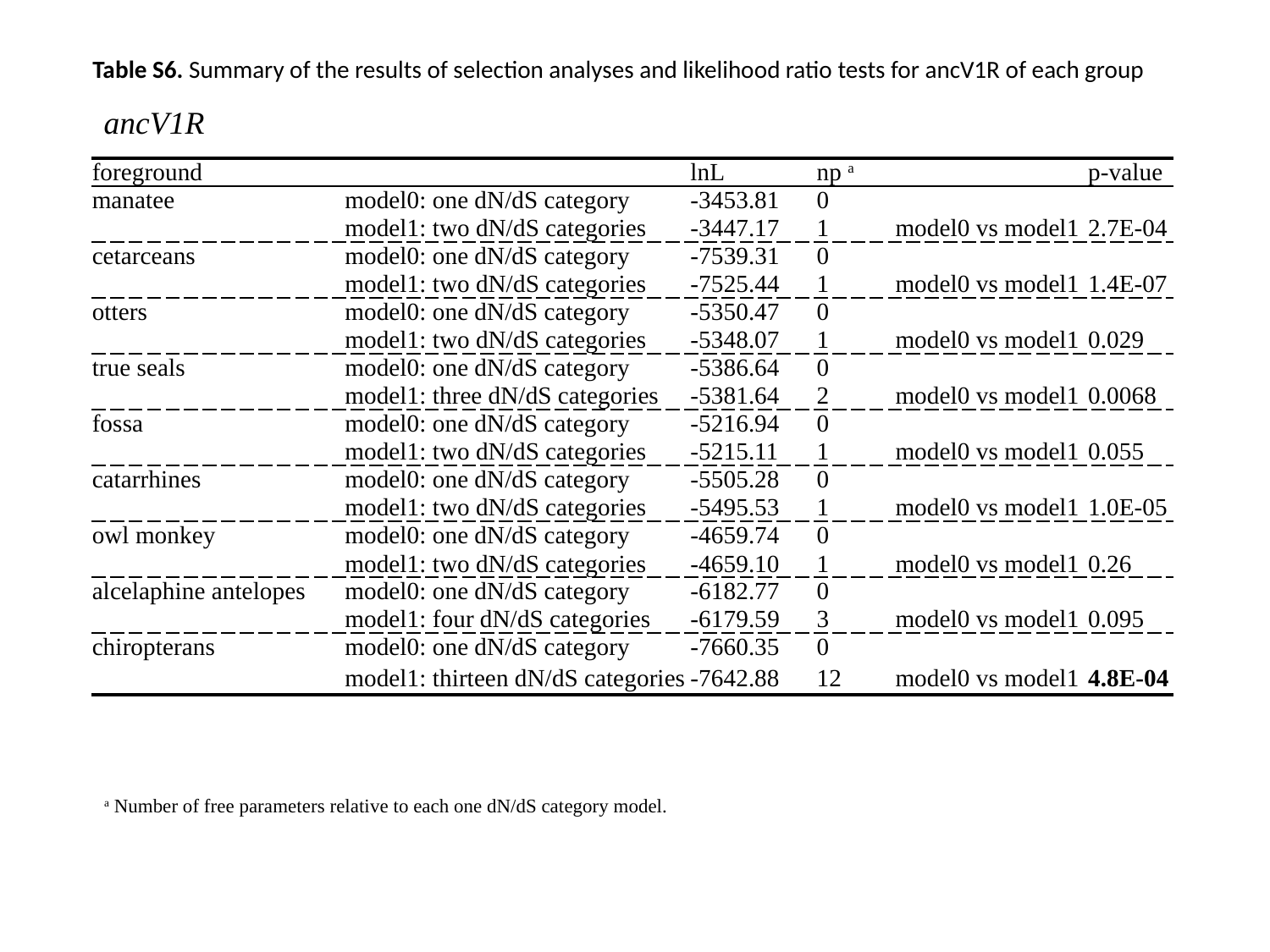

Table S6. Summary of the results of selection analyses and likelihood ratio tests for ancV1R of each group
ancV1R
| foreground | | lnL | np a | | | p-value |
| --- | --- | --- | --- | --- | --- | --- |
| manatee | model0: one dN/dS category | -3453.81 | 0 | | | |
| | model1: two dN/dS categories | -3447.17 | 1 | | model0 vs model1 | 2.7E-04 |
| cetarceans | model0: one dN/dS category | -7539.31 | 0 | | | |
| | model1: two dN/dS categories | -7525.44 | 1 | | model0 vs model1 | 1.4E-07 |
| otters | model0: one dN/dS category | -5350.47 | 0 | | | |
| | model1: two dN/dS categories | -5348.07 | 1 | | model0 vs model1 | 0.029 |
| true seals | model0: one dN/dS category | -5386.64 | 0 | | | |
| | model1: three dN/dS categories | -5381.64 | 2 | | model0 vs model1 | 0.0068 |
| fossa | model0: one dN/dS category | -5216.94 | 0 | | | |
| | model1: two dN/dS categories | -5215.11 | 1 | | model0 vs model1 | 0.055 |
| catarrhines | model0: one dN/dS category | -5505.28 | 0 | | | |
| | model1: two dN/dS categories | -5495.53 | 1 | | model0 vs model1 | 1.0E-05 |
| owl monkey | model0: one dN/dS category | -4659.74 | 0 | | | |
| | model1: two dN/dS categories | -4659.10 | 1 | | model0 vs model1 | 0.26 |
| alcelaphine antelopes | model0: one dN/dS category | -6182.77 | 0 | | | |
| | model1: four dN/dS categories | -6179.59 | 3 | | model0 vs model1 | 0.095 |
| chiropterans | model0: one dN/dS category | -7660.35 | 0 | | | |
| | model1: thirteen dN/dS categories | -7642.88 | 12 | | model0 vs model1 | 4.8E-04 |
a Number of free parameters relative to each one dN/dS category model.

## Slide 6
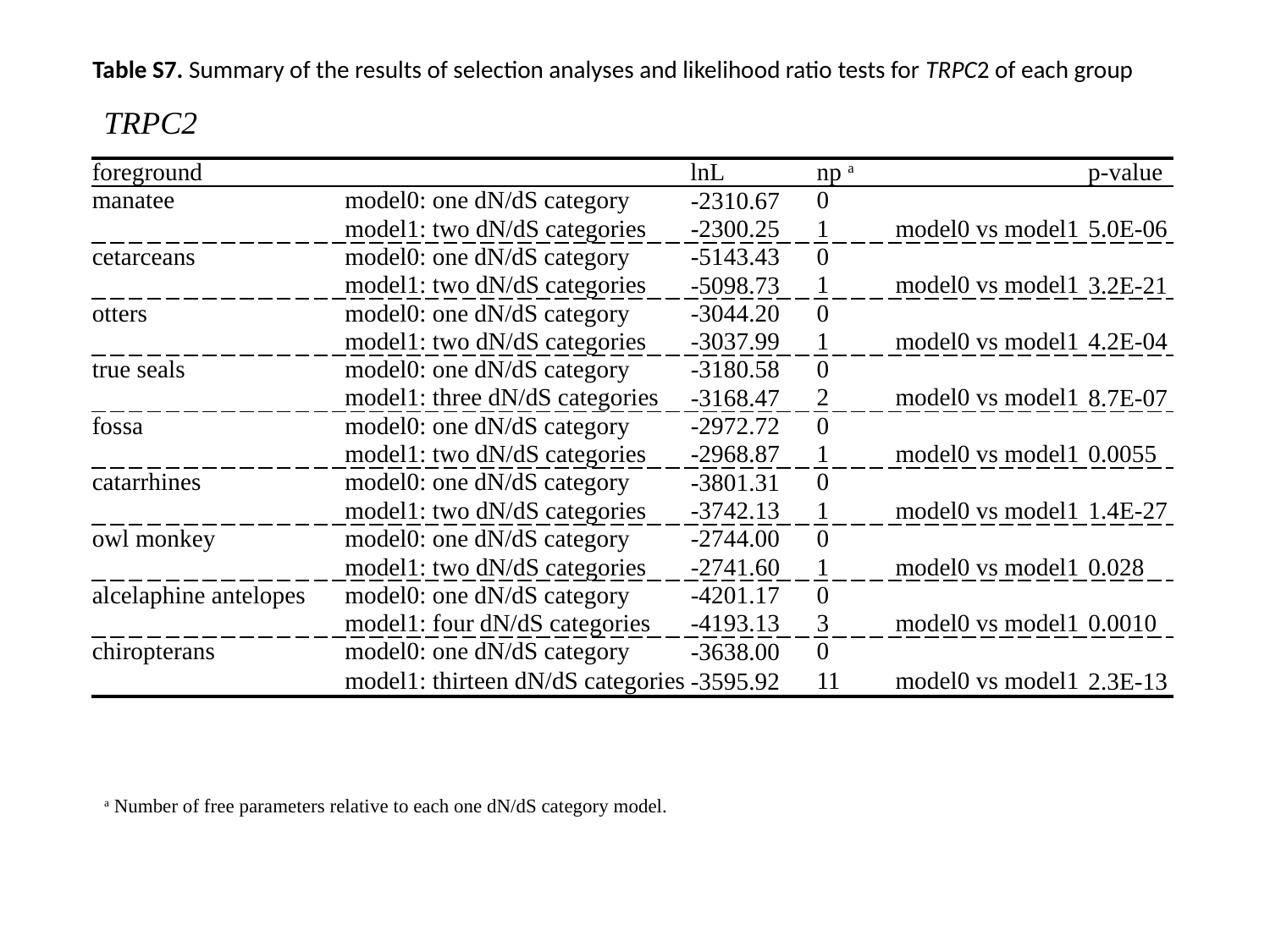

Table S7. Summary of the results of selection analyses and likelihood ratio tests for TRPC2 of each group
TRPC2
| foreground | | lnL | np a | | | p-value |
| --- | --- | --- | --- | --- | --- | --- |
| manatee | model0: one dN/dS category | -2310.67 | 0 | | | |
| | model1: two dN/dS categories | -2300.25 | 1 | | model0 vs model1 | 5.0E-06 |
| cetarceans | model0: one dN/dS category | -5143.43 | 0 | | | |
| | model1: two dN/dS categories | -5098.73 | 1 | | model0 vs model1 | 3.2E-21 |
| otters | model0: one dN/dS category | -3044.20 | 0 | | | |
| | model1: two dN/dS categories | -3037.99 | 1 | | model0 vs model1 | 4.2E-04 |
| true seals | model0: one dN/dS category | -3180.58 | 0 | | | |
| | model1: three dN/dS categories | -3168.47 | 2 | | model0 vs model1 | 8.7E-07 |
| fossa | model0: one dN/dS category | -2972.72 | 0 | | | |
| | model1: two dN/dS categories | -2968.87 | 1 | | model0 vs model1 | 0.0055 |
| catarrhines | model0: one dN/dS category | -3801.31 | 0 | | | |
| | model1: two dN/dS categories | -3742.13 | 1 | | model0 vs model1 | 1.4E-27 |
| owl monkey | model0: one dN/dS category | -2744.00 | 0 | | | |
| | model1: two dN/dS categories | -2741.60 | 1 | | model0 vs model1 | 0.028 |
| alcelaphine antelopes | model0: one dN/dS category | -4201.17 | 0 | | | |
| | model1: four dN/dS categories | -4193.13 | 3 | | model0 vs model1 | 0.0010 |
| chiropterans | model0: one dN/dS category | -3638.00 | 0 | | | |
| | model1: thirteen dN/dS categories | -3595.92 | 11 | | model0 vs model1 | 2.3E-13 |
a Number of free parameters relative to each one dN/dS category model.

## Slide 7
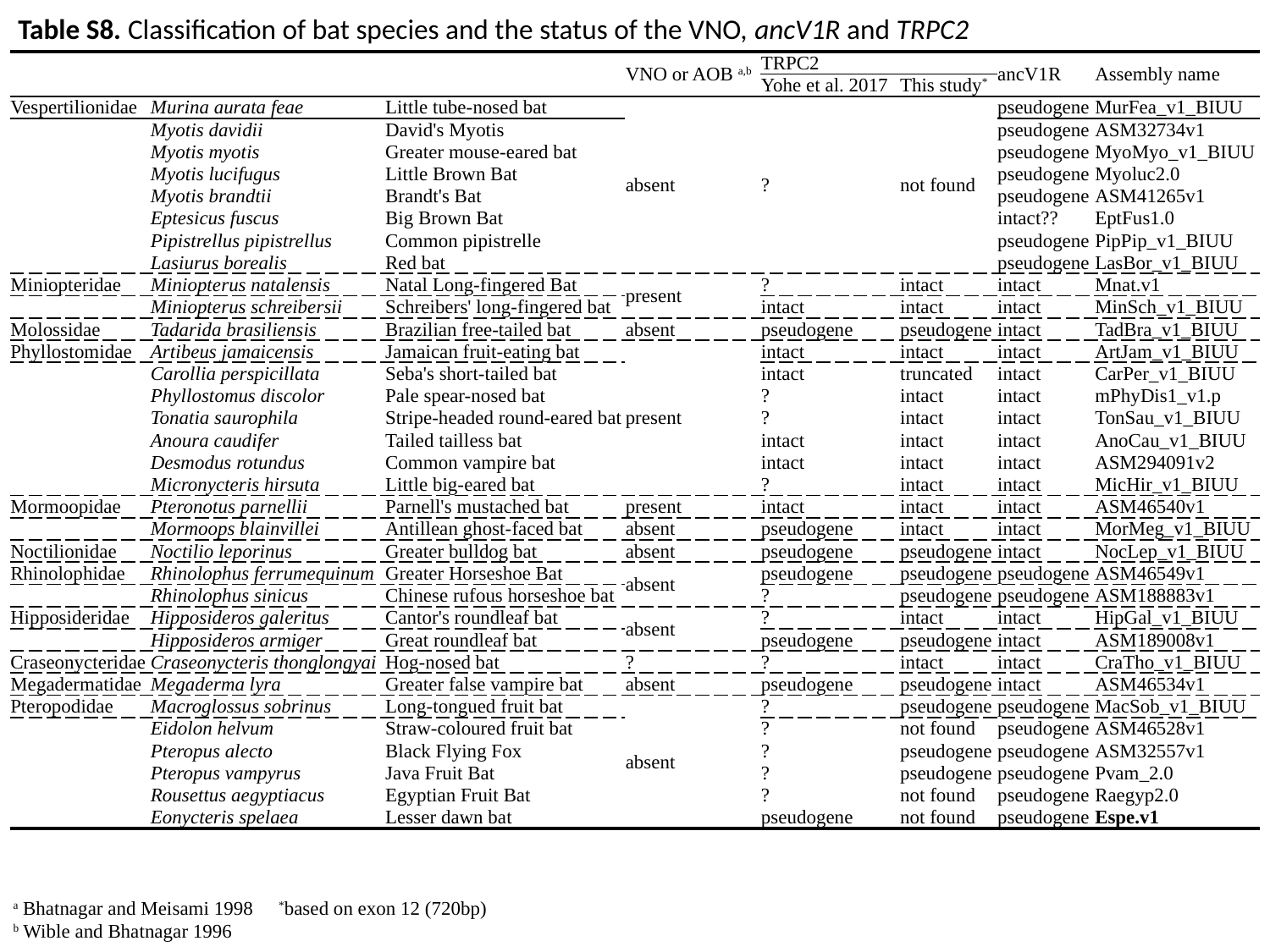

Table S8. Classification of bat species and the status of the VNO, ancV1R and TRPC2
| | | | VNO or AOB a,b | TRPC2 | | ancV1R | Assembly name |
| --- | --- | --- | --- | --- | --- | --- | --- |
| | | | | Yohe et al. 2017 | This study\* | | |
| Vespertilionidae | Murina aurata feae | Little tube-nosed bat | absent | ? | not found | pseudogene | MurFea\_v1\_BIUU |
| | Myotis davidii | David's Myotis | | | | pseudogene | ASM32734v1 |
| | Myotis myotis | Greater mouse-eared bat | | | | pseudogene | MyoMyo\_v1\_BIUU |
| | Myotis lucifugus | Little Brown Bat | | | | pseudogene | Myoluc2.0 |
| | Myotis brandtii | Brandt's Bat | | | | pseudogene | ASM41265v1 |
| | Eptesicus fuscus | Big Brown Bat | | | | intact?? | EptFus1.0 |
| | Pipistrellus pipistrellus | Common pipistrelle | | | | pseudogene | PipPip\_v1\_BIUU |
| | Lasiurus borealis | Red bat | | | | pseudogene | LasBor\_v1\_BIUU |
| Miniopteridae | Miniopterus natalensis | Natal Long-fingered Bat | present | ? | intact | intact | Mnat.v1 |
| | Miniopterus schreibersii | Schreibers' long-fingered bat | | intact | intact | intact | MinSch\_v1\_BIUU |
| ‎Molossidae | Tadarida brasiliensis | Brazilian free-tailed bat | absent | pseudogene | pseudogene | intact | TadBra\_v1\_BIUU |
| Phyllostomidae | Artibeus jamaicensis | Jamaican fruit-eating bat | present | intact | intact | intact | ArtJam\_v1\_BIUU |
| | Carollia perspicillata | Seba's short-tailed bat | | intact | truncated | intact | CarPer\_v1\_BIUU |
| | Phyllostomus discolor | Pale spear-nosed bat | | ? | intact | intact | mPhyDis1\_v1.p |
| | Tonatia saurophila | Stripe-headed round-eared bat | | ? | intact | intact | TonSau\_v1\_BIUU |
| | Anoura caudifer | Tailed tailless bat | | intact | intact | intact | AnoCau\_v1\_BIUU |
| | Desmodus rotundus | Common vampire bat | | intact | intact | intact | ASM294091v2 |
| | Micronycteris hirsuta | Little big-eared bat | | ? | intact | intact | MicHir\_v1\_BIUU |
| Mormoopidae | Pteronotus parnellii | Parnell's mustached bat | present | intact | intact | intact | ASM46540v1 |
| | Mormoops blainvillei | Antillean ghost-faced bat | absent | pseudogene | intact | intact | MorMeg\_v1\_BIUU |
| Noctilionidae | Noctilio leporinus | Greater bulldog bat | absent | pseudogene | pseudogene | intact | NocLep\_v1\_BIUU |
| Rhinolophidae | Rhinolophus ferrumequinum | Greater Horseshoe Bat | absent | pseudogene | pseudogene | pseudogene | ASM46549v1 |
| | Rhinolophus sinicus | Chinese rufous horseshoe bat | | ? | pseudogene | pseudogene | ASM188883v1 |
| Hipposideridae | Hipposideros galeritus | Cantor's roundleaf bat | absent | ? | intact | intact | HipGal\_v1\_BIUU |
| | Hipposideros armiger | Great roundleaf bat | | pseudogene | pseudogene | intact | ASM189008v1 |
| Craseonycteridae | Craseonycteris thonglongyai | Hog-nosed bat | ? | ? | intact | intact | CraTho\_v1\_BIUU |
| Megadermatidae | Megaderma lyra | Greater false vampire bat | absent | pseudogene | pseudogene | intact | ASM46534v1 |
| Pteropodidae | Macroglossus sobrinus | Long-tongued fruit bat | absent | ? | pseudogene | pseudogene | MacSob\_v1\_BIUU |
| | Eidolon helvum | Straw-coloured fruit bat | | ? | not found | pseudogene | ASM46528v1 |
| | Pteropus alecto | Black Flying Fox | | ? | pseudogene | pseudogene | ASM32557v1 |
| | Pteropus vampyrus | Java Fruit Bat | | ? | pseudogene | pseudogene | Pvam\_2.0 |
| | Rousettus aegyptiacus | Egyptian Fruit Bat | | ? | not found | pseudogene | Raegyp2.0 |
| | Eonycteris spelaea | Lesser dawn bat | | | | | |
| | | | | pseudogene | not found | pseudogene | Espe.v1 |
a Bhatnagar and Meisami 1998
b Wible and Bhatnagar 1996
*based on exon 12 (720bp)

## Slide 8
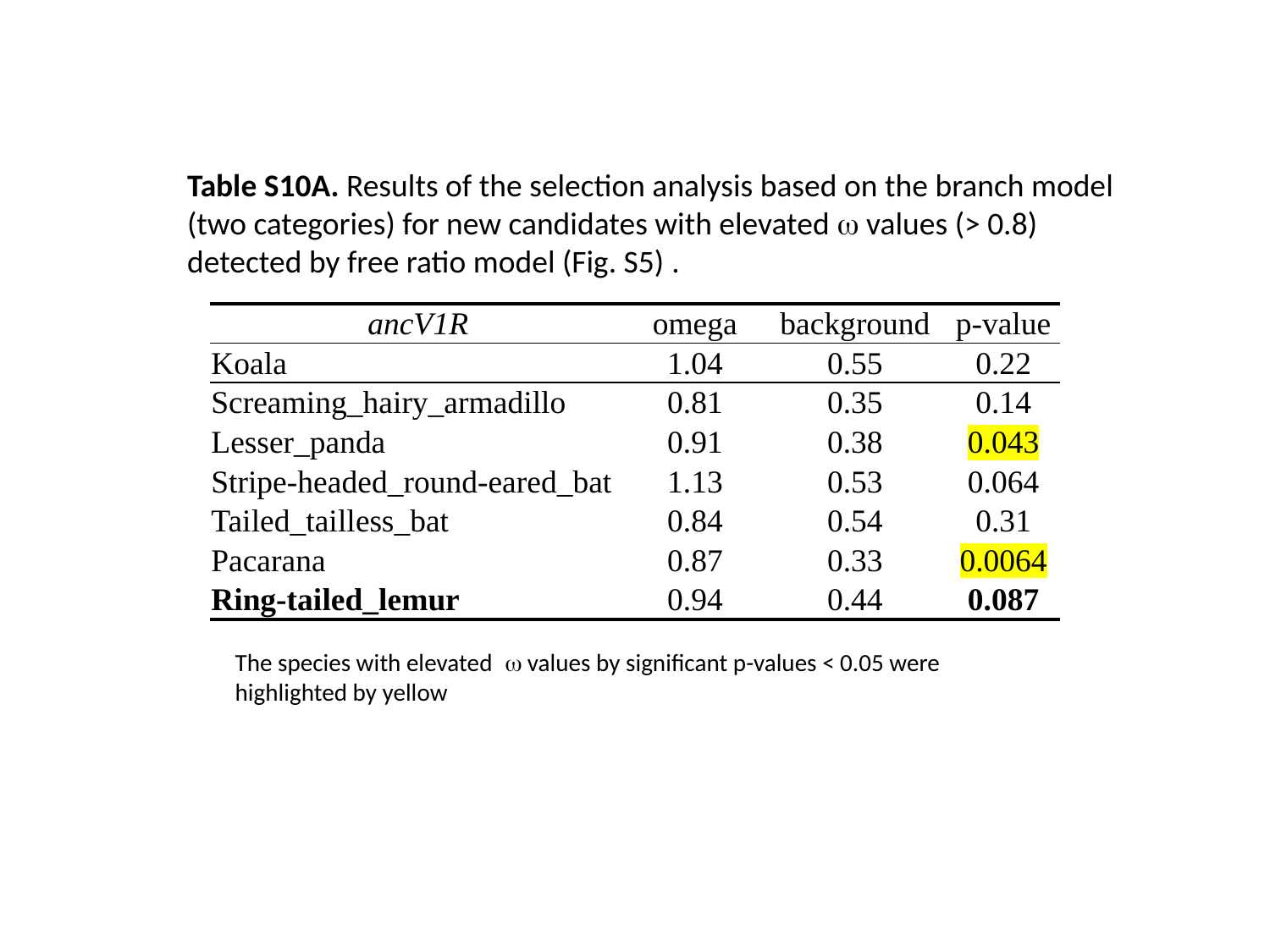

Table S10A. Results of the selection analysis based on the branch model (two categories) for new candidates with elevated w values (> 0.8) detected by free ratio model (Fig. S5) .
| ancV1R | omega | background | p-value |
| --- | --- | --- | --- |
| Koala | 1.04 | 0.55 | 0.22 |
| Screaming\_hairy\_armadillo | 0.81 | 0.35 | 0.14 |
| Lesser\_panda | 0.91 | 0.38 | 0.043 |
| Stripe-headed\_round-eared\_bat | 1.13 | 0.53 | 0.064 |
| Tailed\_tailless\_bat | 0.84 | 0.54 | 0.31 |
| Pacarana | 0.87 | 0.33 | 0.0064 |
| Ring-tailed\_lemur | 0.94 | 0.44 | 0.087 |
The species with elevated w values by significant p-values < 0.05 were highlighted by yellow

## Slide 9
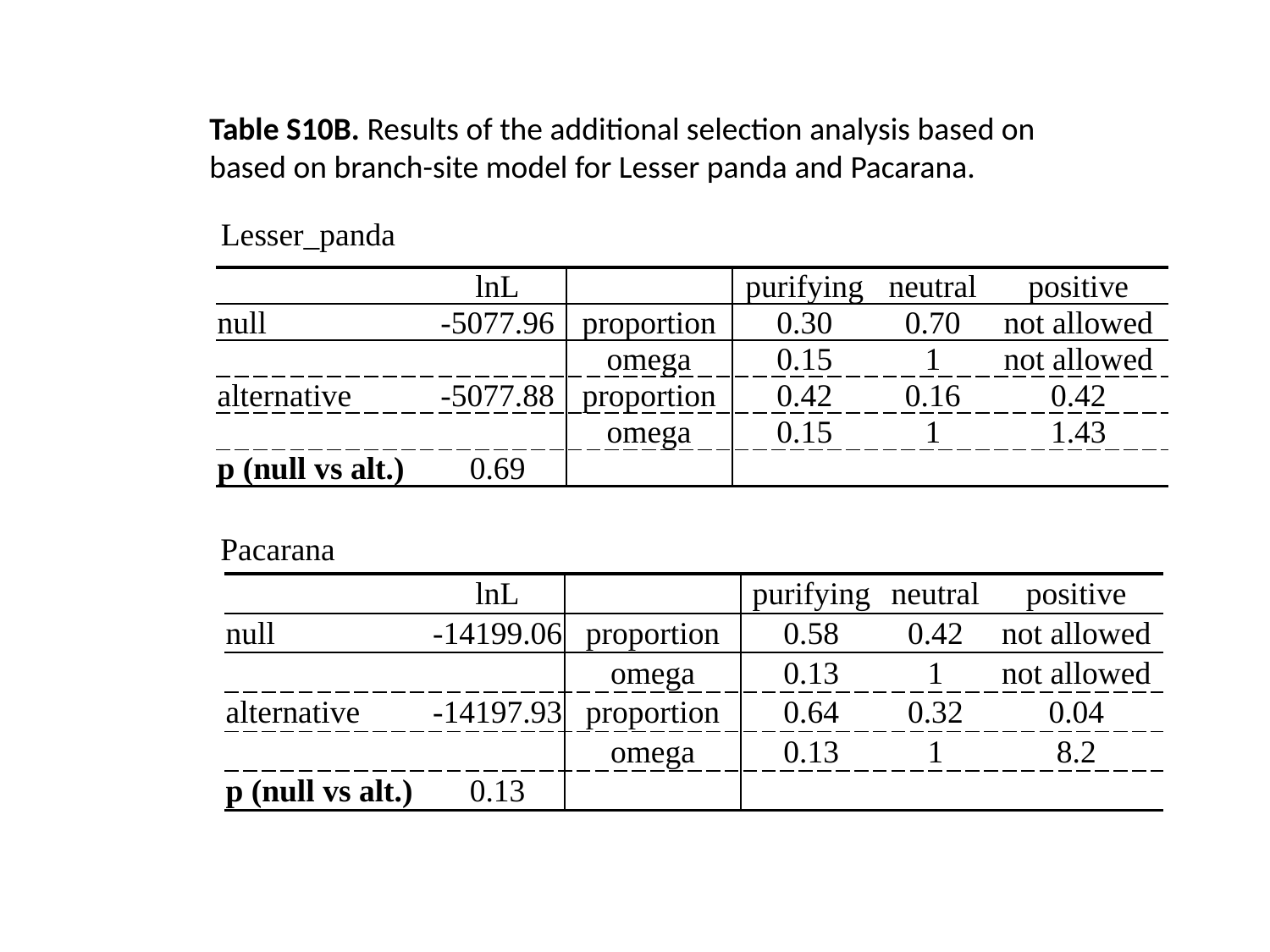

Table S10B. Results of the additional selection analysis based on based on branch-site model for Lesser panda and Pacarana.
Lesser_panda
| | lnL | | purifying | neutral | positive |
| --- | --- | --- | --- | --- | --- |
| null | -5077.96 | proportion | 0.30 | 0.70 | not allowed |
| | | omega | 0.15 | 1 | not allowed |
| alternative | -5077.88 | proportion | 0.42 | 0.16 | 0.42 |
| | | omega | 0.15 | 1 | 1.43 |
| p (null vs alt.) | 0.69 | | | | |
Pacarana
| | lnL | | purifying | neutral | positive |
| --- | --- | --- | --- | --- | --- |
| null | -14199.06 | proportion | 0.58 | 0.42 | not allowed |
| | | omega | 0.13 | 1 | not allowed |
| alternative | -14197.93 | proportion | 0.64 | 0.32 | 0.04 |
| | | omega | 0.13 | 1 | 8.2 |
| p (null vs alt.) | 0.13 | | | | |
