# Supplementary material for: Inactivation of ancV1R as a Predictive Signature for the Loss of Vomeronasal System in Mammals
Source: Genome Biol Evol. 2020 Apr 21;12(6):766–78. doi: 10.1093/gbe/evaa082 (PMC7290294; doi:10.1093/gbe/evaa082)
Supplement: evaa082_Supplementary_Data [file evaa082_supplementary_data.zip › Table S9.pptx]

## Slide 1
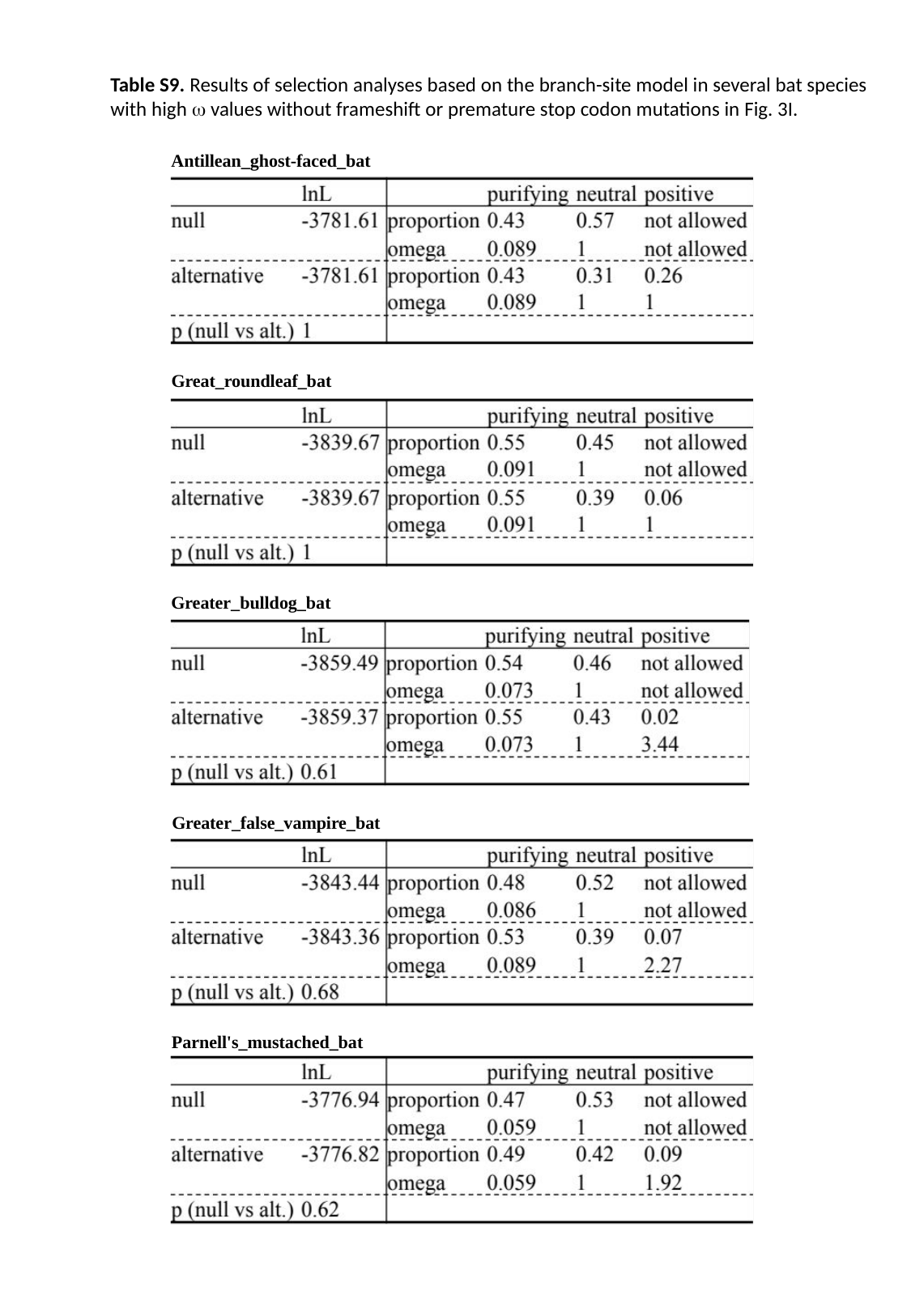

Table S9. Results of selection analyses based on the branch-site model in several bat species with high w values without frameshift or premature stop codon mutations in Fig. 3I.
Antillean_ghost-faced_bat
Great_roundleaf_bat
Greater_bulldog_bat
Greater_false_vampire_bat
Parnell's_mustached_bat
