# Supplementary figures and images for: Inactivation of ancV1R as a Predictive Signature for the Loss of Vomeronasal System in Mammals
Source: Genome Biol Evol. 2020 Apr 21;12(6):766–78. doi: 10.1093/gbe/evaa082 (PMC7290294; doi:10.1093/gbe/evaa082)

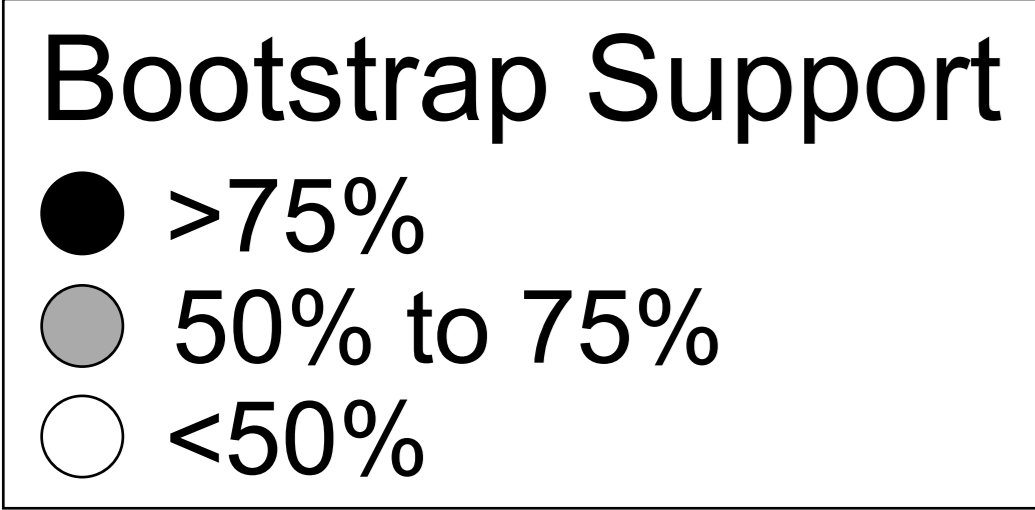

Supplement: evaa082_Supplementary_Data [file evaa082_supplementary_data.zip › Supplementary Fig S1.pdf]

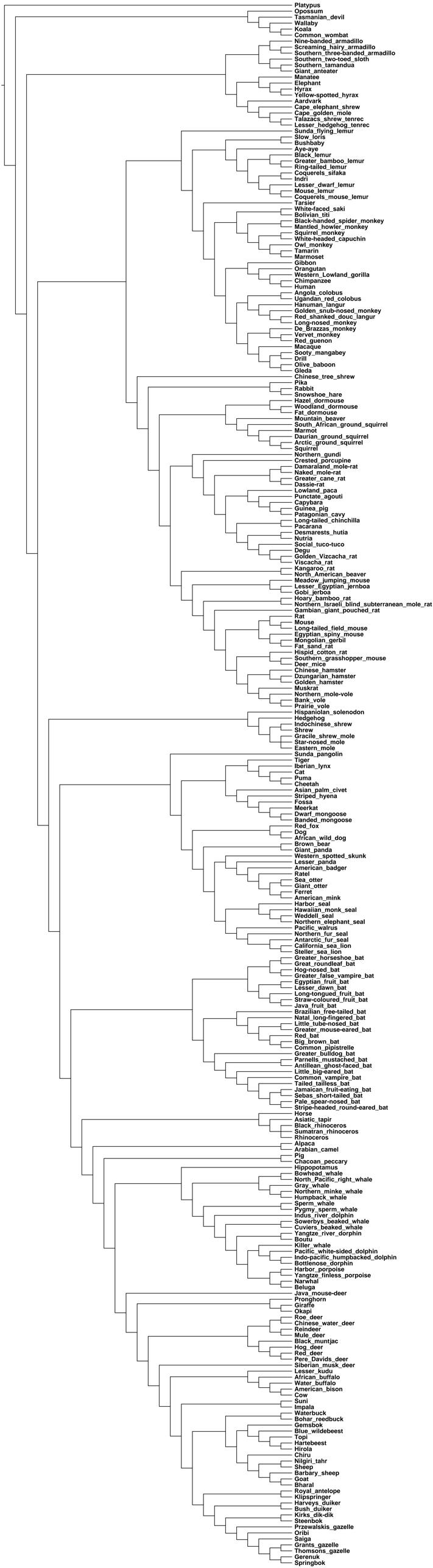

Supplement: evaa082_Supplementary_Data [file evaa082_supplementary_data.zip › Supplementary Fig S2.pdf]

## Slide 1
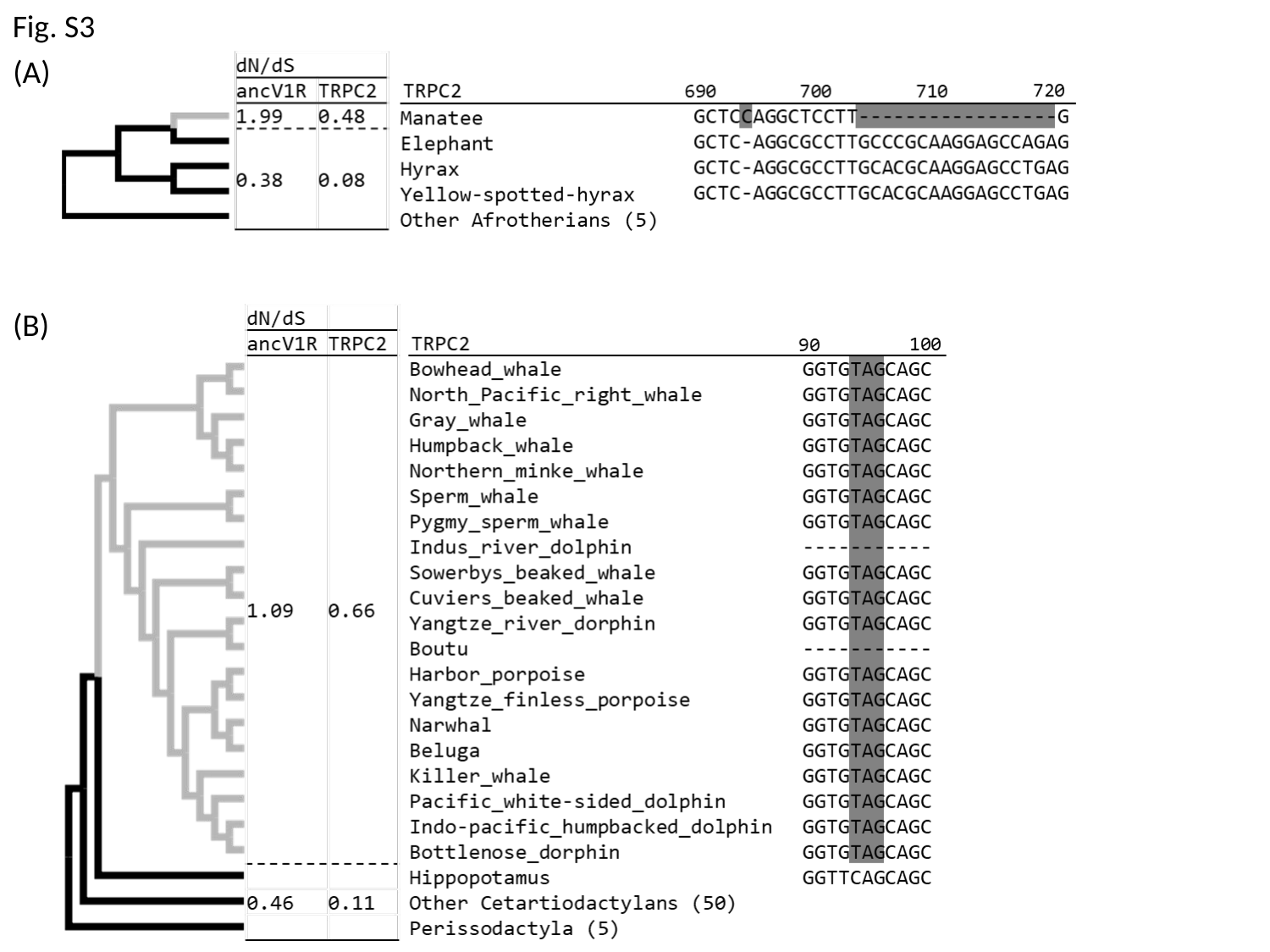

Fig. S3
(A)
(B)

## Slide 2
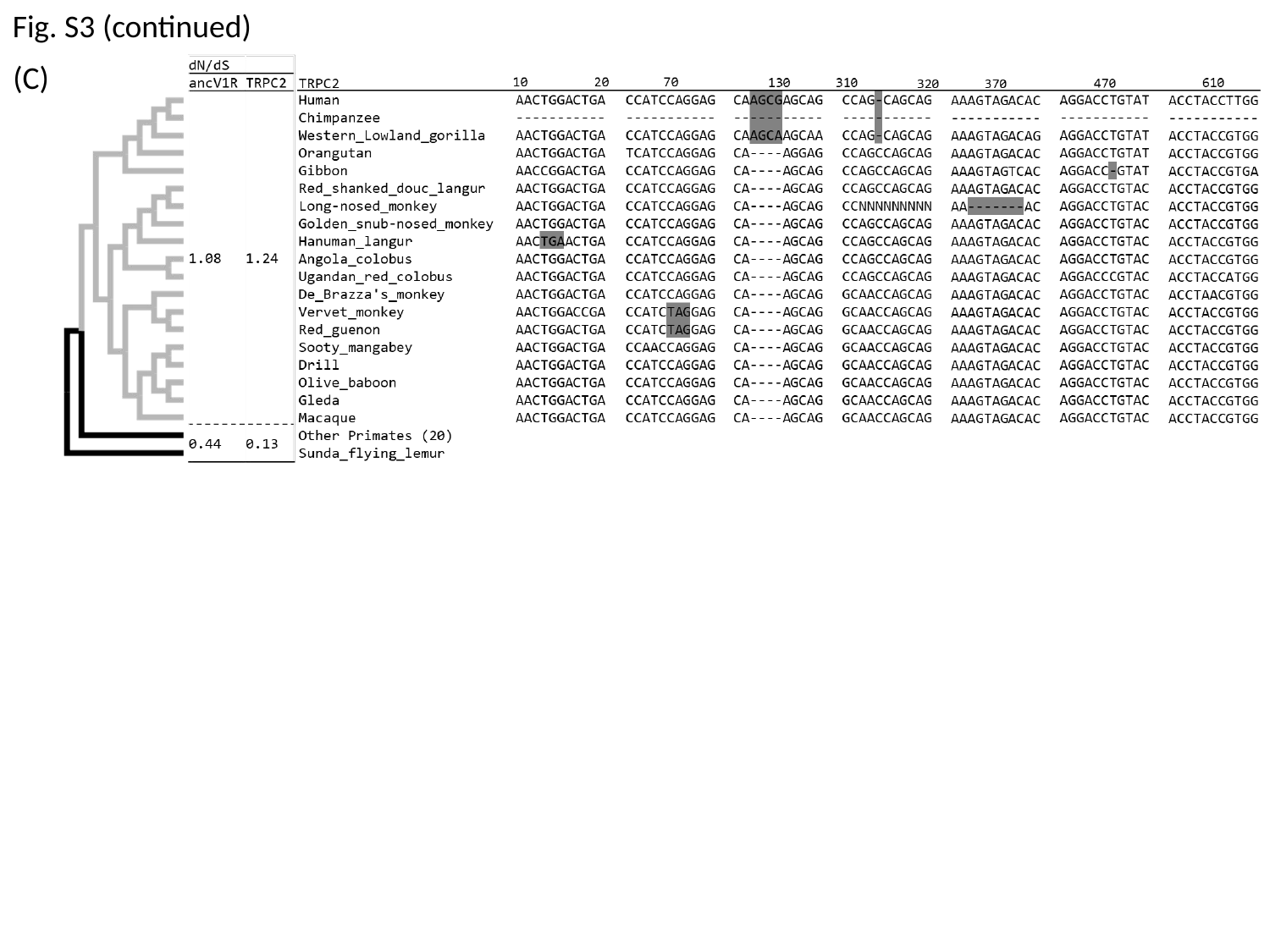

Fig. S3 (continued)
(C)

## Slide 3
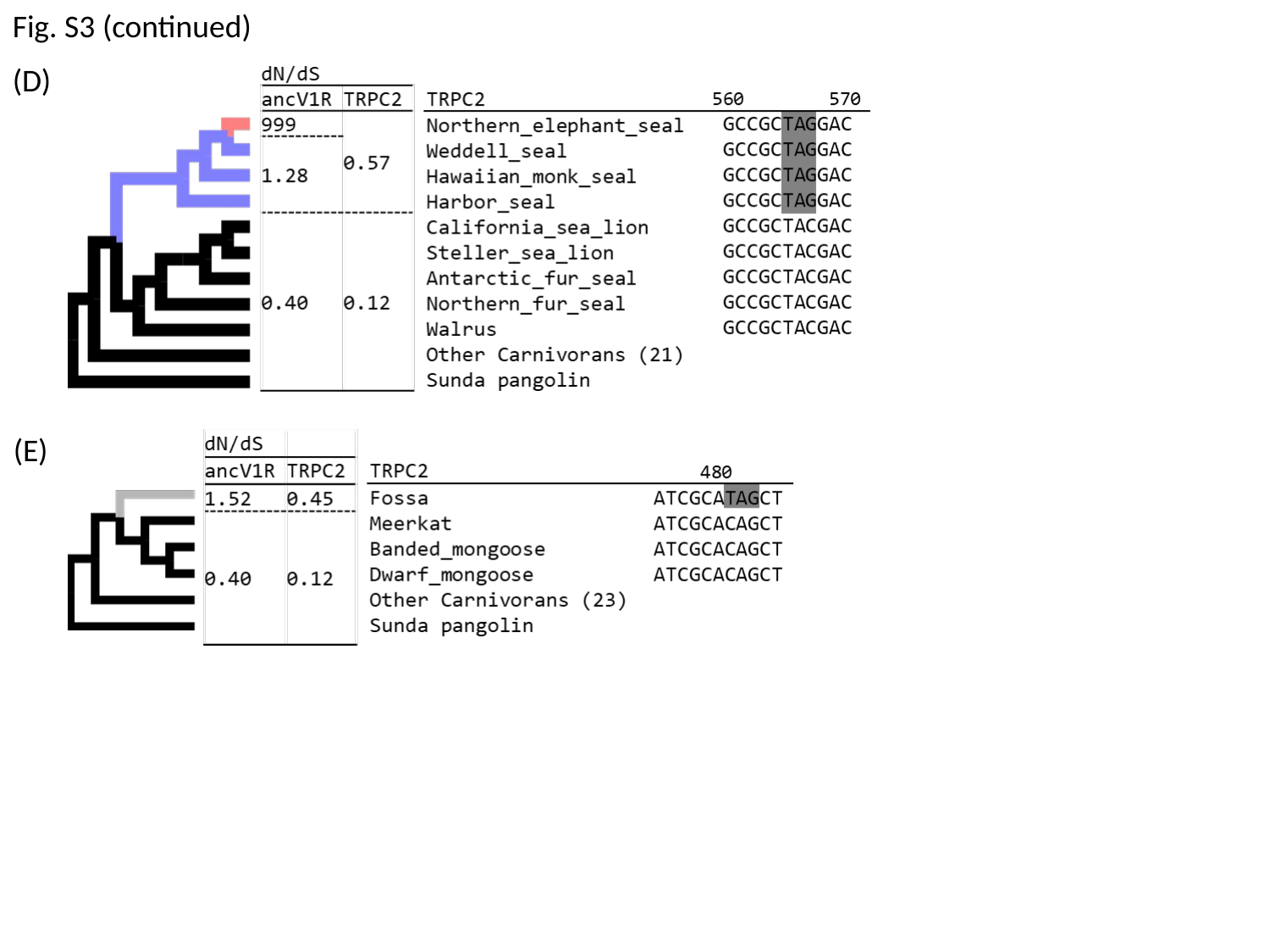

Fig. S3 (continued)
(D)
(E)

## Slide 4
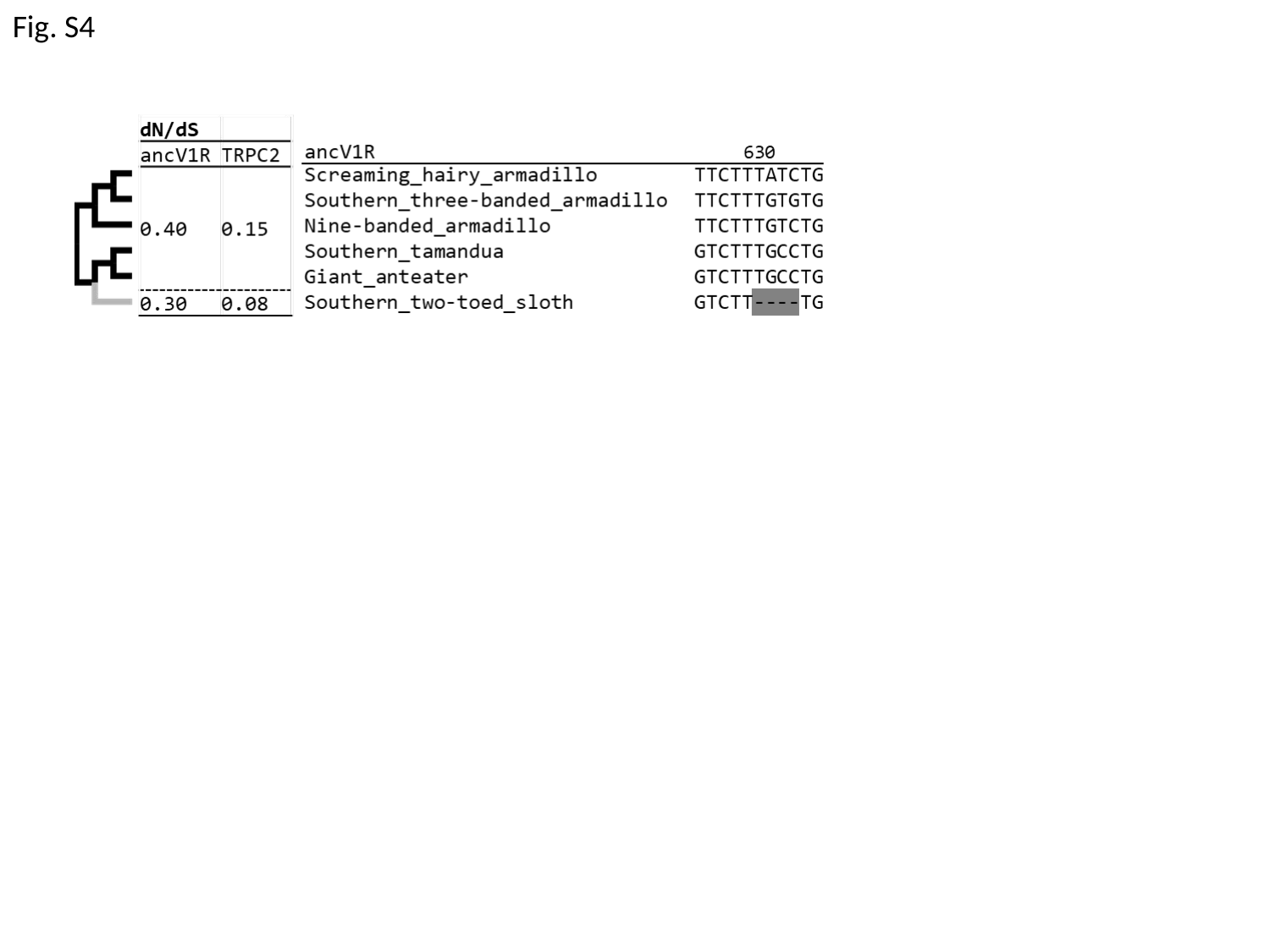

Fig. S4

Supplement: evaa082_Supplementary_Data [file evaa082_supplementary_data.zip › Supplementary Fig S3-S4.pptx]

A

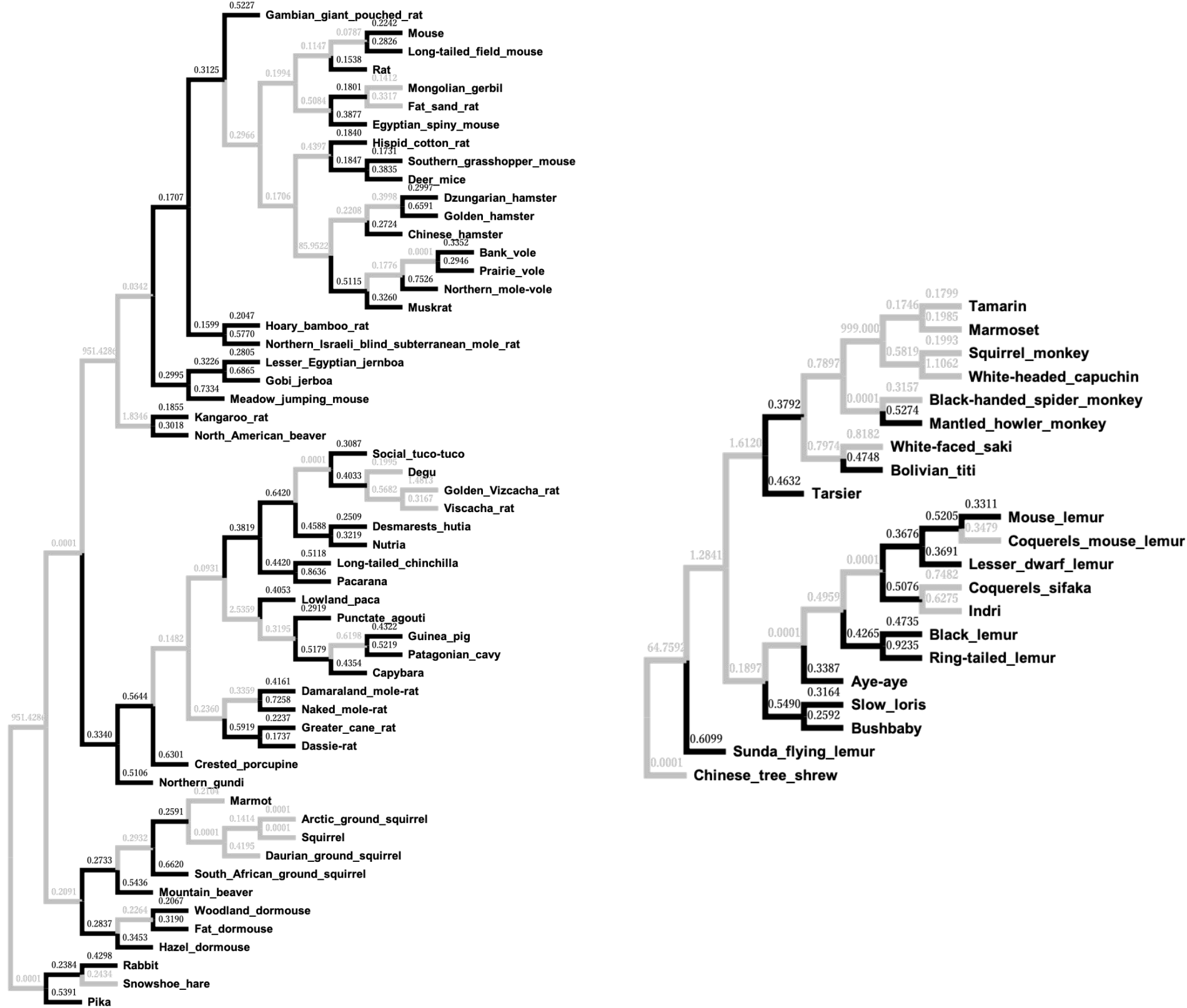

B

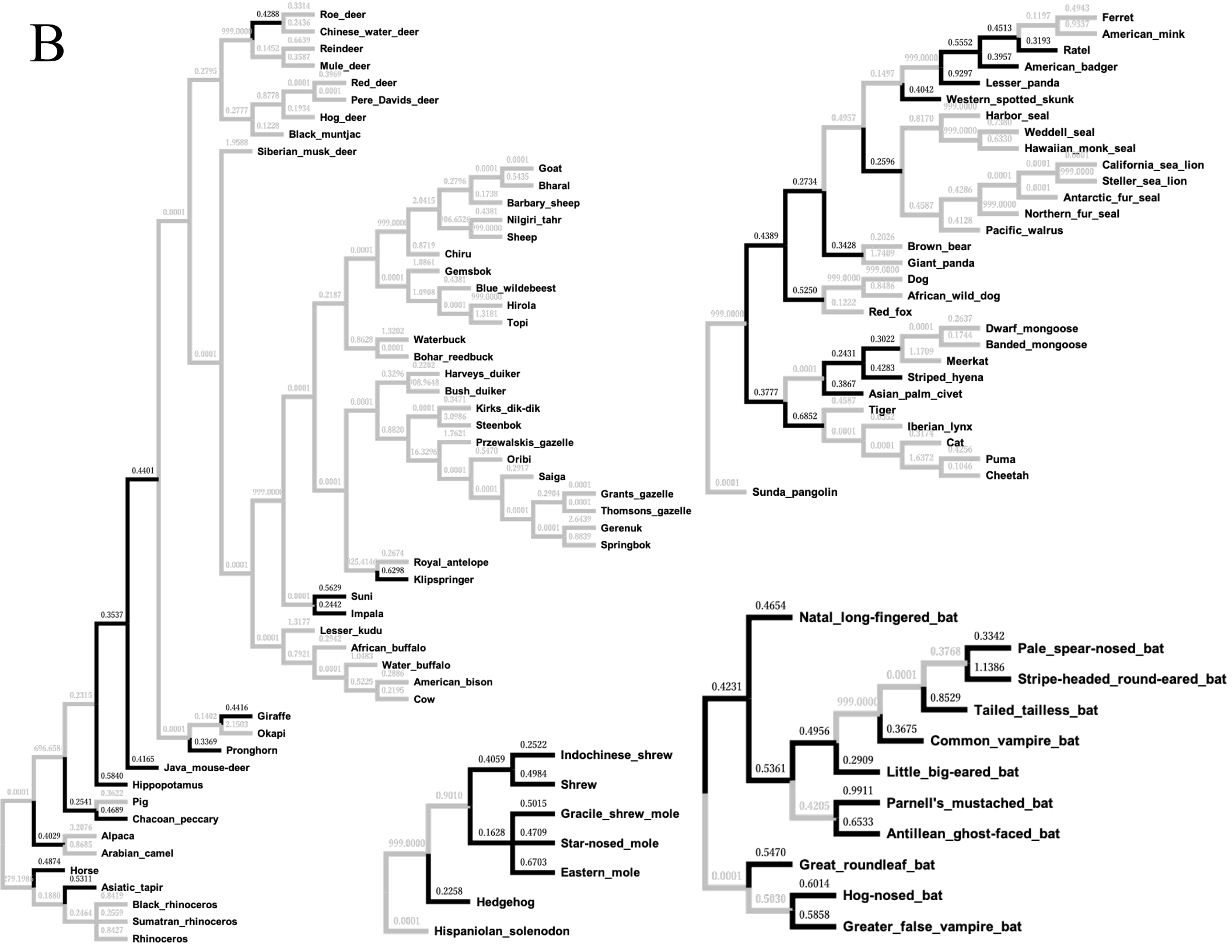

C

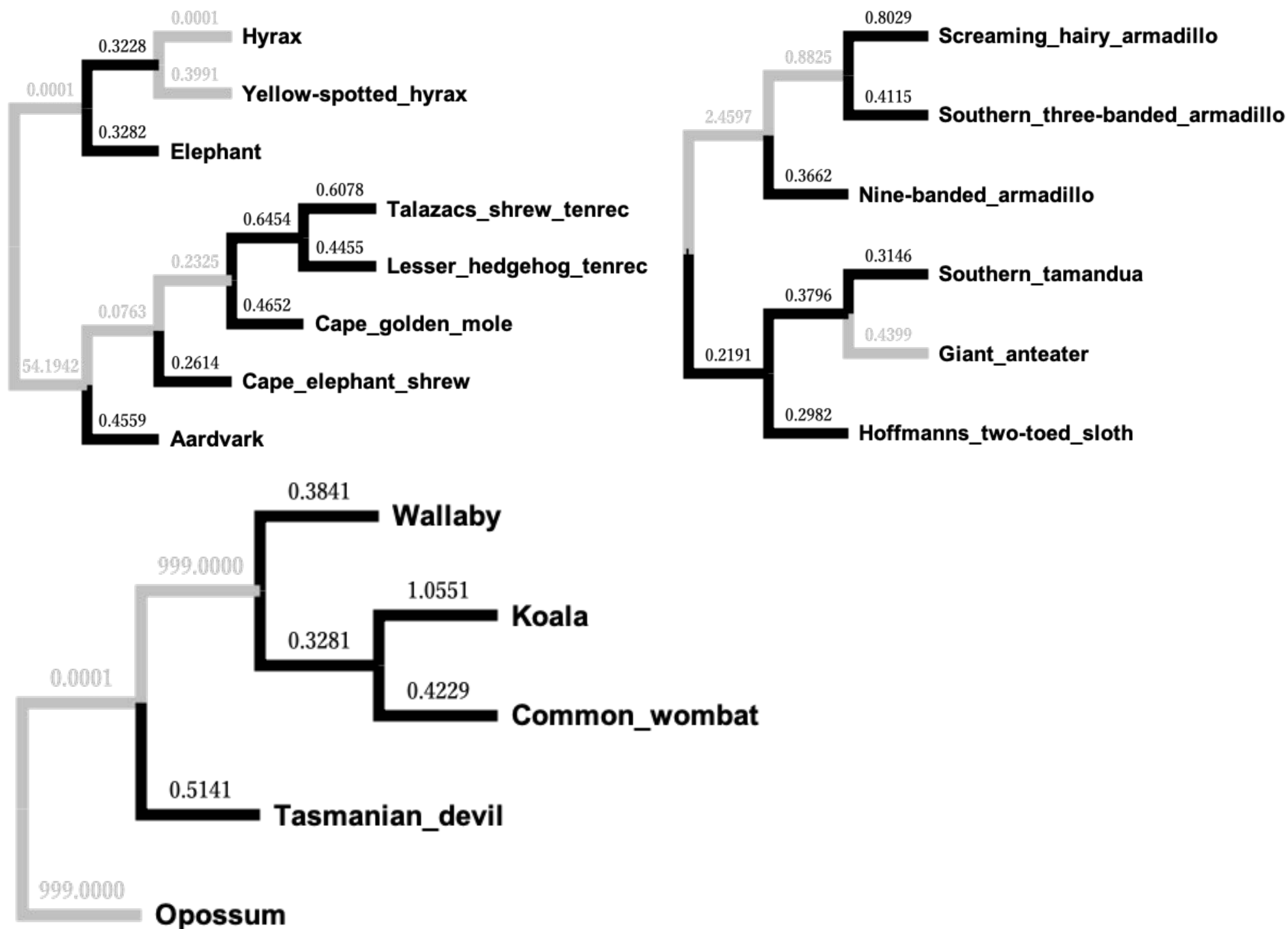

Supplement: evaa082_Supplementary_Data [file evaa082_supplementary_data.zip › Supplementary_FigS5.pdf]
